# Histamine in Brazilian Foods: A Comprehensive Review of Occurrence and Risk Assessment for Intoxication and Intolerance

**DOI:** 10.1002/fsn3.71151

**Published:** 2025-11-17

**Authors:** Maria Beatriz A. Gloria, Fabiana B. Diniz, Bruno M. Dala‐Paula, Biane Philadelpho, José Eduardo Gonçalves, Ederlan S. Ferreira, Livia Simon Sarkadi

**Affiliations:** ^1^ Programa de Pós‐Graduação em Ciência de Alimentos, Faculdade de Farmácia UFMG Belo Horizonte Brazil; ^2^ Programa de Pós‐Graduação em Ciência de Alimentos, Faculdade de Farmácia Universidade Federal da Bahia Salvador Brazil; ^3^ Laboratório de Controle de Qualidade, Departamento de Produtos Farmacêuticos, Faculdade de Farmácia UFMG Belo Horizonte Brazil; ^4^ Programa de Pós‐Graduação em Nutrição e Longevidade Universidade Federal de Alfenas, Alfenas, UNIFAL Alfenas Brazil; ^5^ Hungarian University of Agriculture and Life Sciences Budapest Hungary

**Keywords:** analysis, biogenic amines, exposure, health effects, histamine‐free diet, safety

## Abstract

Even though histamine plays a vital role in human health, it has been associated with food intoxication and intolerance, accompanied by gastrointestinal, neurological, cardiovascular, respiratory, and dermatological symptoms. In this way, one must be aware of foods containing histamine to minimize exposure, which can be achieved by following low‐ or histamine‐free diets. Therefore, reliable information regarding histamine in foods is required. The most relevant, current state of knowledge regarding histamine's biochemical and health aspects is provided. A systematic review of histamine in Brazilian food was conducted from 1980 to 2024. One hundred fifty‐six studies were available, and the histamine levels were obtained for 17 food groups, including 343 foods. The most widely used analytical method was HPLC with post‐column derivatization (*o*‐phthalaldehyde) and fluorometric detection. In most studies, nine other amines were quantified simultaneously besides histamine. Most animal‐origin products had histamine. Histamine levels in dry‐fermented sausages, aged and grated cheeses, scombroid fish, and crabs reached values capable of causing histamine intoxication. Foods of plant origin had lower histamine. However, some foods, including eggplant, soy sauce, wine, and sprouts, had higher levels. Moreover, although not enough to elicit intoxication, they can contribute to the total histamine in a meal. Several foods must be avoided to prevent histamine intolerance. However, some foods did not contain detectable histamine. The detailed histamine information provided allows individuals with histamine intolerance and healthcare professionals to make confident food choices, which avoids restrictive diets and improves the quality of life.

## Graphical abstract

Even though histamine plays an important role in human health, it has been associated with food intoxication and intolerance episodes. The most relevant, current state of knowledge regarding histamine's biochemical and health aspects is provided. Histamine levels are provided for 17 food groups, including 335 food types and 11,437 samples, allowing individuals with histamine intolerance to make confident food choices, avoid restrictive diets, and improve their quality of life.

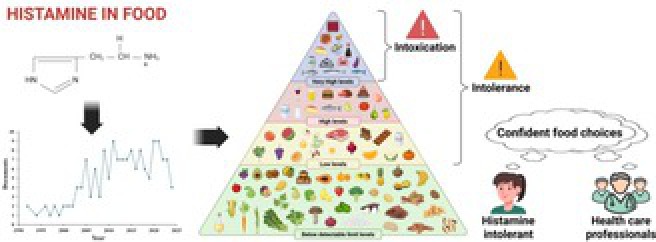

**Abbreviations**
CAD: cadaverine
DAO: diaminoxidase
EFSA: European Food Safety Authority
HIM: histamine
LOQ: limit of quantification
MAO: monoaminoxidase
MAOI: monoaminoxidase inhibitor
NOAEL: no adverse effect level
PUT: putrescine

## 1 Introduction

Histamine (HIM) stands out as one of the most intriguing bioactive amines, as it is relevant from both food quality and safety points of view (Gloria 2005; Ruiz‐Capillas and Herrero 2019; Simon Sarkadi 2019; Ding and Li 2024). Even though it is naturally present in some foods, HIM can be formed and accumulate in food due to inadequate hygienic‐sanitary conditions or deficient temperature control during food production, processing, and storage (Ruiz‐Capillas and Herrero 2019; Simon Sarkadi 2019; Schirone, Esposito, et al. 2022). In addition, HIM can be formed and can accumulate during fermentation, due to the free histidine decarboxylating enzyme activity of starter and non‐starter microorganisms (Ferrante and Mercogliano 2023; Turna et al. 2024). HIM is also relevant to human health. Even though required at low levels for important physiological activities, e.g., neurotransmission, vasodilation, and immune responses (Dala‐Paula et al. 2023; Wang et al. 2024), HIM, at high levels, is associated with outbreaks of HIM intoxication (EFSA 2017; FAO/WHO 2018; Colombo et al. 2018; Zhernov et al. 2023). However, recently, HIM intolerance, a disorder in HIM homeostasis that increases plasma levels mainly due to reduced diamine oxidase (DAO) activity, is increasingly being reported (Zingone et al. 2023; Jochum 2024). The primary strategy for preventing the occurrence of HIM intolerance is to consume a HIM‐free or low‐HIM diet. These diets have gained popularity through social media; however, scientific data supporting their use are lacking. In addition, there is no consensus regarding which foods should be included in these diets. Furthermore, there are scarce scientific compilations of HIM levels in food (Ordóñez et al. 2016; Sánchez‐Pérez et al. 2021).

The objective of this study was to provide relevant, up‐to‐date information on HIM biochemistry and its effects on human health, to compile scientific data through a systematic literature review from 1980 to 2024 on the occurrence of HIM in Brazilian food, and to assess the risk of HIM intoxication and HIM intolerance. This review provides detailed lists of HIM in Brazilian food products of animal and plant origins. It includes a list of products that should be avoided to prevent HIM intoxication and intolerance.

## 2 The Chemistry of Histamine

HIM was first isolated from the plant fungus ergot in 1910 by English scientists Henry Dale and George Barger, and in 1911, they isolated the substance from animal tissues (Tiligada and Ennis 2020). HIM (CAS: 51–45‐6; PubChem CID: 5818) is the common name of 2‐(1H‐imidazol‐5‐yl) ethanamine (IUPAC), or 1H‐Imidazole‐4‐ethanamine. It has the molecular formula C_5_H_9_N_3_ and a molecular mass of 111.15 g/mol. It is solid at room temperature, with a melting point of 83°C–84°C and a boiling point of 209°C–210°C. It has a pKa1 of 6.04. HIM is a member of the imidazole class, e.g., 1H‐imidazole is substituted at C‐4 with a 2‐aminoethyl group. It is a heterocyclic diamine originating from histidine decarboxylation. Its common name is derived from histidine, its precursor amino acid (Gloria 2005; Tiligada and Ennis 2020).

## 3 Physiological Importance of Histamine

HIM is a powerful, biologically active compound with many vital roles. It is synthesized and stored in secretory granules, mainly in mast cells and basophils, as well as in gastric enterochromaffin cells, lymph nodes, thymus, and histaminergic neurons (Tiligada and Ennis 2020; Hrubisko et al. 2021). It elicits multifaceted modulatory functions by activating four receptors. Histamine H1 receptors promote blood vessel dilation (potent capillary dilator leading to hypotensive effects), airway constriction, and itching. H2 receptors regulate gastric acid secretion. H3 receptors modulate the sleep–wake rhythm, whereas H4 receptors influence the immune system. The release of HIM and its effects are tightly regulated at the cellular and local tissue levels. Histamine in mast cells and basophils is a major mediator of IgE and non‐IgE‐mediated immunological responses (Panula 2021; Dala‐Paula et al. 2023). It can directly stimulate the heart and cause contraction or relaxation of the extravascular smooth muscles. HIM can stimulate sensory and motor neurons. It also mediates primary and immediate symptoms of allergic responses (Panula 2021; Dala‐Paula et al. 2023; Wang et al. 2024). The beneficial effects of histamine on human health are indicated in Table 1. Accordingly, HIM plays essential roles in numerous human pathophysiological processes.

**TABLE 1:** The beneficial effects of histamine on human health.

Beneficial effects	References
Cardiovascular (promotion of vascular permeability and vasodilatation)	Kovacova‐Hanuskova et al. (2015); Worm et al. (2019); Moya‐García et al. (2021); Comas‐Basté et al. (2020)
Circadian cycle regulation	Simons and Simons (2011); Moya‐García et al. (2021)
Stimulation of gastric acid secretion; mediation of intestinal disorders	Kovacova‐Hanuskova et al. (2015); Worm et al. (2019); Tiligada and Ennis (2020); Comas‐Basté et al. (2020); Smolinska et al. (2022)
Mediation of inflammatory response: increase of the release of other mediators from mast cells and basophils, downregulate humoral immunity; and upregulate T helper‐1 (TH‐1) priming, TH‐1 proliferation, interferon‐gamma production, cellular adhesion molecule expression, and chemotaxis of eosinophils and neutrophils	Simons and Simons (2011); Worm et al. (2019); Tiligada and Ennis (2020); Comas‐Basté et al. (2020); Shulpekova et al. (2021); Wang et al. (2024)
Smooth muscle cell contraction (particularly the bronchi and intestine)	Kovacova‐Hanuskova et al. (2015); Worm et al. (2019); Comas‐Basté et al. (2020)
Regulation of cell proliferation and differentiation, hematopoiesis, embryonic development, regeneration, and wound healing	Simons and Simons (2011); Kovacova‐Hanuskova et al. (2015)
Neurotransmission: synthesized by neurons located in the posterior region of the hypothalamus, whose axons extend through the brain	Worm et al. (2019); Tiligada and Ennis (2020)
Anticonvulsant activity; contribution to the regulation of vigilance (alertness and attention), cognition, learning, and memory	Simons and Simons (2011); Thakkar (2011)

## 4 Histamine Metabolism

**FIGURE 1:**
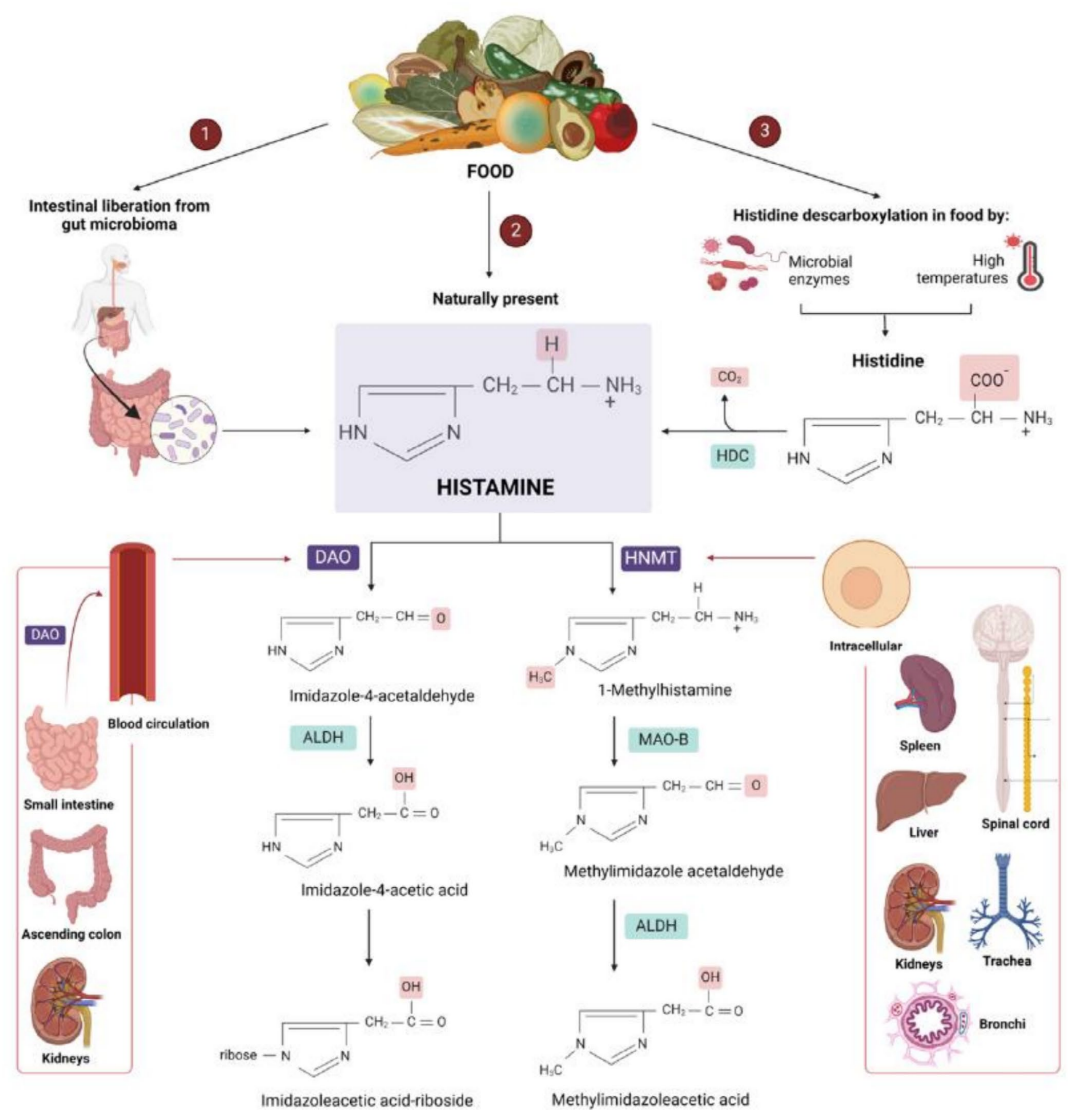
Pathways for the formation of histamine in food and its metabolism in the human body. Histamine can be naturally present in food [❷], liberated from the gut microbiota [❶] or it can be formed by histidine decarboxylase (HDC) activity [❸]. Histamine is metabolized either by diamine oxidase (DAO) [❷‐DAO], or by histamine‐N‐methyltransferase (HNMT) [❷‐HNMT]. In the first case [❷‐DAO], DAO catalyzes the oxidative deamination of histamine, producing imidazole‐4‐acetaldehyde, ammonia, and hydrogen peroxide. Imidazole‐4‐acetaldehyde is further oxidized by an aldehyde dehydrogenase (ALDH), leading to the formation of imidazole‐4‐acetic acid, which can be excreted, or it can be converted to the riboside form. During histamine metabolization by HNMT [❷‐HNMT], histamine undergoes methylation, producing 1‐methylhistamine, which is further metabolized by monoamine oxidase‐B (MAO‐B), producing methylimidazole acetaldehyde and then methylimidazole acetic acid by ALDH, which can be excreted.

Besides the synthesis in the human body, HIM is found in food. It can be naturally present in food or formed during food production, processing, and storage. It is primarily formed through microbial enzymes’ decarboxylation of free histidine (Figure 1). Free histidine is available in food and can be liberated during protein hydrolysis. Microorganisms can be naturally present in food (inherent microbiota). They can be added as starter cultures in fermented products or contaminate the food when inadequate sanitary and hygienic conditions are prevalent (Gloria 2005; Ruiz‐Capillas and Herrero 2019; Moniente et al. 2021). HIM can also be formed during intense heat treatments, by thermal histidine decarboxylation (Macheiner et al. 2022). Following ingestion, HIM reaches the intestine and passes through the intestinal epithelium. It is metabolized in two pathways. The main path converts HIM to imidazole acetic acid via oxidative degradation by DAO, a secretory enzyme present in most body tissues but mainly in the small intestinal mucosa and kidneys (Figure 1). The second pathway involves histamine‐N‐methyltransferase (HNMT) methylation, producing methylhistamine, which is then metabolized by monoamine oxidase‐B (MAO‐B) to produce methylimidazole acetic acid. DAO is responsible for inactivating extracellular HIM, whereas HNMT can only convert HIM within the intracellular space. HNMT is highly selective for HIM, whereas DAO can metabolize other diamines such as putrescine (PUT), cadaverine (CAD), and agmatine (Sánchez‐Pérez et al. 2022; Dala‐Paula et al. 2023; Zingone et al. 2023).

## 5 Adverse Effects Associated With Histamine

Healthy individuals rapidly detoxify dietary HIM. However, when the availability of HIM is high or its degradation mechanism is impaired, there can be health disorders, including HIM intoxication and intolerance.

Several factors can prevent HIM degradation. It can be impaired by reduced DAO activity due to genetic (polymorphism), pathological (intestinal and other diseases), or pharmacological factors (Comas‐Basté et al. 2019; Hrubisko et al. 2021). Intestinal diseases, including small bowel pathologies, can impair DAO activity (Zingone et al. 2023). DAO deficiency is correlated with the degree of mucosal damage, intestinal permeability, malabsorption of carbohydrates, non‐coeliac gluten sensitivity, and dysbiosis of the intestinal microbiota (Sánchez‐Pérez et al. 2022; Zingone et al. 2023). Copper and C, B1, and B6 vitamins deficiencies can also decrease DAO activity (Shulpekova et al. 2021). DAO activity can also be affected by the menstrual cycle (Hrubisko et al. 2021) and by some diseases, including migraines, atopic dermatitis, irritable bowel syndrome, cyclic vomiting syndrome, and muscular pain (Worm et al. 2019; Hrubisko et al. 2021).

In addition, DAO can be inhibited by xenobiotics or drugs, increasing the risk of HIM's adverse effects. Drugs with DAO inhibitory activity include antibiotics and antihypertensives (Comas‐Basté et al. 2020; Dala‐Paula et al. 2023; Rentzos et al. 2024). This effect may be temporary and can be reversed by discontinuing the DAO‐blocking medications (Zingone et al. 2023). Another possible factor affecting HIM metabolism is the concomitant presence of other diamines in food, including PUT and CAD (EFSA 2011; Sánchez‐Pérez et al. 2022). However, limited experimental evidence supports this hypothesis (Zingone et al. 2023).

### 5.1 Histamine Intoxication

HIM intoxication, also called HIM poisoning or scombroid food poisoning, arises from the ingestion of histamine‐rich foods. The symptoms typically emerge within minutes to a few hours after food ingestion, i.e., it has a short incubation period (EFSA 2011; Dala‐Paula et al. 2023). It occurs as an outbreak of low to moderate severity, lasting only a few hours. The most common symptoms are described in Table 2. They are associated with the physiological role of histamine in the body, affecting the skin, the gastrointestinal tract, and some hemodynamic and neurological aspects (EFSA 2011, 2017; FAO/WHO 2018; Hrubisko et al. 2021). However, life‐threatening anaphylactic reactions can occur, depending on the amount of HIM ingested and the individual's sensitivity to HIM (EFSA 2011; FAO/WHO 2018; Yu et al. 2018; Comas‐Basté et al. 2020; Hungerford 2021).

**TABLE 2:** Adverse effects of histamine on human health and associated symptoms.

Adverse effects on human health	References
*Histamine intoxication (histamine or scombroid poisoning)*	
Typically lasts up to 24 h, producing allergy‐like symptoms	Colombo et al. (2018); Hungerford (2021); Zhernov et al. (2023); Zingone et al. (2023)
Respiratory: bronchial obstruction, respiratory distress, bronchospasm, cough, suffocation	Kovacova‐Hanuskova et al. (2015); Shulpekova et al. (2021); Zhernov et al. (2023)
Hemodynamic: hypotension	Zingone et al. (2023)
Gastrointestinal: oral burning, metallic taste, nausea, vomiting, diarrhea, intestinal ischemia	Kovacova‐Hanuskova et al. (2015)
Skin: redness, edema (eyelids), hives, urticaria, rash, itching, pruritus, erythema, swelling (face, tongue, eyelids), localized inflammation	Hrubisko et al. (2021); Kovacova‐Hanuskova et al. (2015); Zingone et al. (2023)
Neurological: headache, palpitation, flushing, tingling, burning, and itching; dizziness, loss of consciousness	Kovacova‐Hanuskova et al. (2015); Worm et al. (2019); Zhernov et al. (2023); Zingone et al. (2023)
Cardiovascular: tachycardia, hypo‐ and hypertension	Kovacova‐Hanuskova et al. (2015); Zhernov et al. (2023); Zingone et al. (2023)
*Histamine intolerance (non‐allergic food intolerance, histaminosis)*	
Neurological: dizziness, headache	Hrubisko et al. (2021); Shulpekova et al. (2021); Zingone et al. (2023); Jochum (2024)
Respiratory: asthma, sneezing, runny nose, rhinitis, bronchoconstriction, nasal congestion, and breathing difficulty	Reese et al. (2021); Schnedl et al. (2019); Hrubisko et al. (2021); Zingone et al. (2023); Jochum (2024)
Skin: erythema in the facial area, eczema, hives, swelling, flushing, pruritus, itching, urticaria, body rash	Schnedl et al. (2019); Hrubisko et al. (2021); Sánchez‐Pérez et al. (2022); Zingone et al. (2023); Jochum (2024)
Cardiovascular: low blood pressure (subsequent counter‐regulatory hypertension), tachycardia	Reese et al. (2021); Comas‐Basté et al. (2020); Hrubisko et al. (2021); Zingone et al. (2023); Jochum (2024)
Gastrointestinal: nausea, vomiting, diarrhea, constipation, abdominal pain, distension, postprandial fullness	Reese et al. (2021); Schnedl and Enko (2021); Zingone et al. (2023); Jochum (2024)
Cardiovascular: low blood pressure, tachycardia, hypotonia, and collapse	Reese et al. (2021); Schnedl et al. (2019); Hrubisko et al. (2021); Schnedl and Enko (2021); Sánchez‐Pérez et al. (2022); Zingone et al. (2023); Jochum (2024)

Diagnosis of HIM intoxication is difficult due to the similarity between the symptoms of HIM intoxication and allergies (Kovacova‐Hanuskova et al. 2015; Cheong et al. 2023; Zhernov et al. 2023). However, in HIM intoxication, there is usually a lack of previous history of allergic reactions to the incriminated food. There is often the involvement of more than one individual in group outbreaks. In addition, a particular causative food is identified based on high HIM levels in suspected foods and elevated plasma HIM levels (Comas‐Basté et al. 2020).

### 5.2 Histamine Intolerance

One additional health problem associated with HIM, which has increased in prevalence, is HIM intolerance, also called histaminosis or sensitivity to dietary HIM (Hrubisko et al. 2021; Sánchez‐Pérez et al. 2021). It is described as a non‐immunological condition resulting from an imbalance between histamine uptake from the diet and the capacity to metabolize ingested histamine, leading to increased blood HIM concentration and, therefore, adverse effects (Zingone et al. 2023). In this case, the amount of HIM capable of causing adverse effects is extremely low.

Clinical manifestations of HIM intolerance include a wide range of nonspecific gastrointestinal, neurological, cardiovascular, respiratory, and skin‐related symptoms (Table 2). This is due to the ubiquitous distribution of the four histamine receptors in different body organs and tissues (Comas‐Basté et al. 2020). According to Schnedl et al. (2019), the most frequent symptoms experienced by 133 patients diagnosed with HIM intolerance were gastrointestinal, followed by neurological, cardiovascular, respiratory, and skin‐related. Combinations of three or more symptoms involving different organs were recorded in 97% of the cases, with an average of 11 symptoms per patient. Van Odijk et al. (2023) and Zingone et al. (2023) also observed the prevalence of gastrointestinal symptoms. Histamine intolerance can also be characterized by the symptom stages’ frequencies, timelines, and patterns (Bhattacharjee et al. 2024). The symptoms are milder in HIM intolerance compared to HIM intoxication; however, they often overlap, which complicates differential diagnosis (Dala‐Paula et al. 2023). Diagnosing HIM intolerance is also tricky because of the complexity, low specificity, variable nature of the symptoms, and the similarity of some symptoms with allergies, irritable bowel syndrome, and migraines. This makes it difficult to agree on diagnostic criteria, increasing the risk of misdiagnosis or missed (Comas‐Basté et al. 2020; Bhattacharjee et al. 2024; Tamasi and Kalabay 2025). There are no specific tests that can be used to determine HIM intolerance. However, some potential markers have been suggested, including serum reduced or insufficient DAO activity, skin prick test, HIM challenge test, fecal HIM levels, and genetic testing (Van Odijk et al. 2023; Jochum 2024). So far, no validated diagnostic methods exist for its diagnosis (Zingone et al. 2023).

### 5.3 Threshold Histamine Level for Adverse Health Effects

The critical HIM levels capable of causing histamine intoxication and intolerance are challenging to determine. Regarding HIM intoxication, EFSA (2011) suggested a no adverse effect level (NOAEL) in healthy individuals of 50 mg HIM per person per meal. At that time, a potential acute reference dose (ARfD) of 50 mg of histamine per healthy person was suggested. Colombo et al. (2018), in a systematic literature review and meta‐analysis, assessed HIM levels in food implicated in intoxication episodes worldwide from 1959 to 2013. One hundred three incidents were selected, mainly associated with tuna or Istiophoridae fish species (98%), followed by cheese (2%). The mean HIM level in the incriminated foods was 1107.21 mg/kg, with a confidence interval of 422.69–2900.78 mg/kg. Based on this study, HIM levels ≥ 423 mg/kg could lead to HIM intoxication.

However, for individuals with HIM intolerance, even food with small amounts of HIM may cause adverse health effects. According to EFSA (2011), HIM intolerance can be observed even after exposure to HIM levels below detectable limits in individuals with HIM intolerance.

## 6 Systematic Review on Histamine in Brazilian Food

**FIGURE 2:**
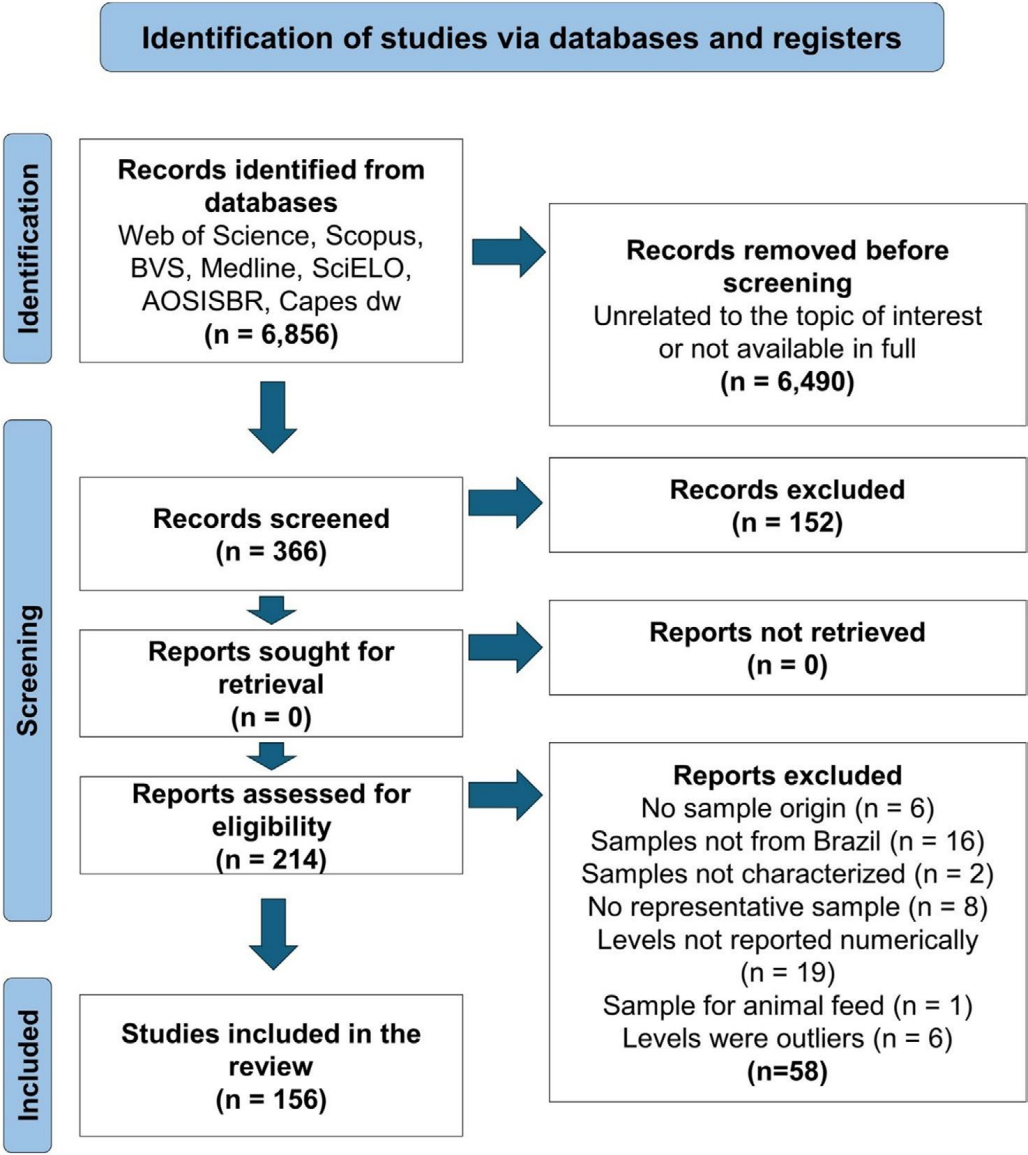
PRIMA flow diagram for the systematic review on the occurrence of histamine in Brazilian food. BVS—virtual health library, SciELO—Latin American digital library, BDTD—Brazilian digital library of theses and dissertations (https://bdtd.ibict.br/vufind/), AOSISBR (https://oasisbr.ibict.br/vufind/), and the CAPES (Coordenação de Aperfeiçoamento de Pessoal de Nível Superior) DW catalog (http://capesdw.capes.gov.br/) Page et al. (2021).

A systematic literature review, following the Preferred Reporting Items for Systematic Reviews and Meta‐Analysis (PRISMA) protocol (Page et al. 2021), collected information from 1980 to 2024 from several databases, as indicated in Figure 2. The search strings were related to the question: “What are the levels of HIM in Brazilian foods?” and included the terms “Food Analysis” OR “Food Composition” AND “Biogenic Amines” OR “Histamine” OR “Bioactive Amines”. After the search, a preliminary selection of documents was made using titles and abstracts. Duplicates were removed, and documents were downloaded for detailed analysis. Some documents were excluded because they did not comply with the eligibility criteria described in Figure 2. The selected documents were used for critical analysis and HIM data extraction. Descriptive statistical analysis was used to determine the evolution of the studies over the years, the food types and groups, the purpose of the studies, and the methods used in the HIM analysis. The levels of HIM in foods were compiled.

### 6.1 Number of Studies Over the Years

**FIGURE 3:**
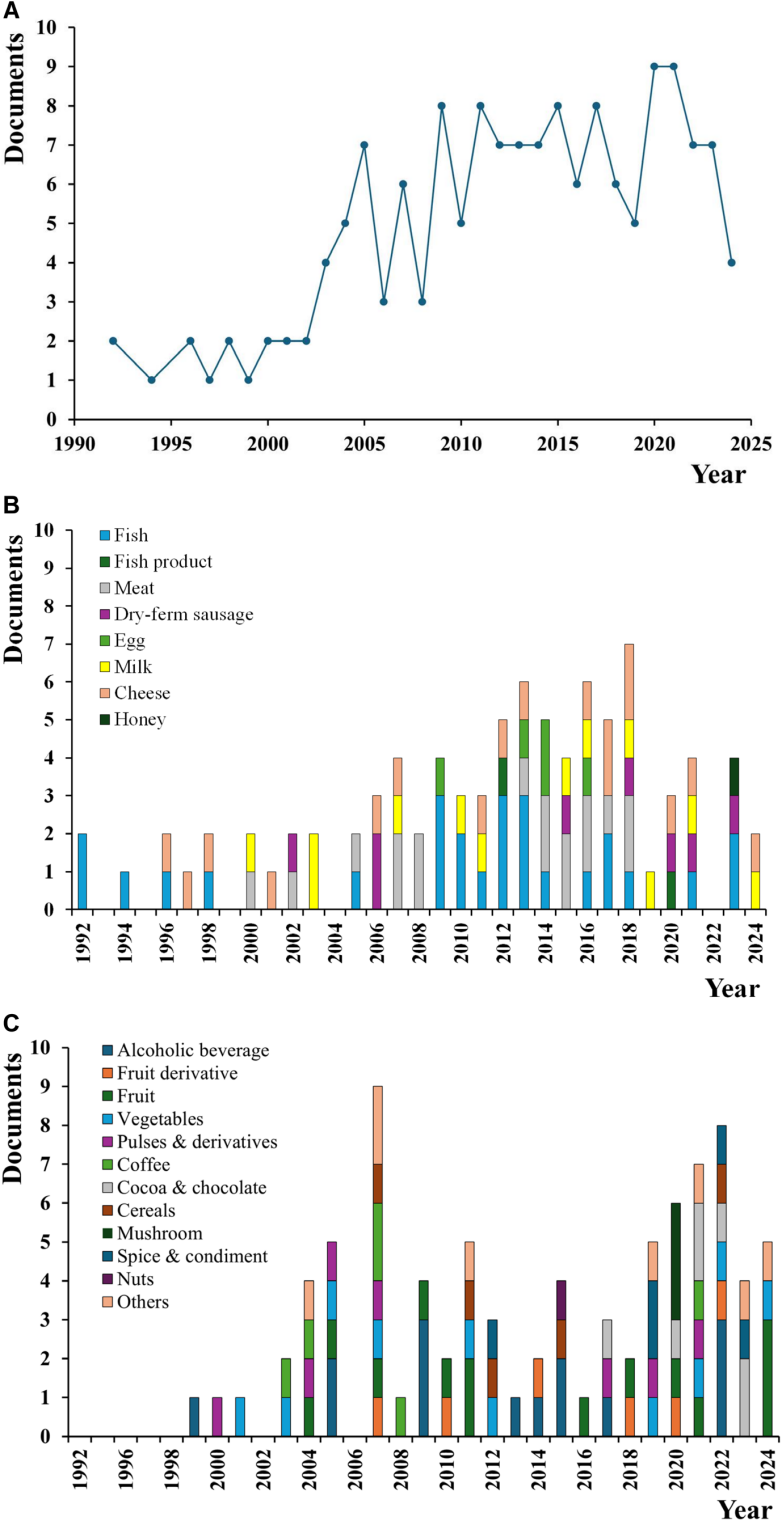
Number of documents containing information on the contents of histamine in Brazilian food from 1992 up to 2024. (A) The year of publication, and by type of food group, e.g., (B) animal origin, and (C) plant origin. Dry‐ferm sausage—dry‐fermented sausage. Others: Miscellaneous (oil, sweets, seaweed, extracts).

The systematic literature search identified an initial pool of 6856 documents published between 1980 and 2024, but only 156 papers were selected and included in this study (Figure 2). Because no information was available from 1980 to 1991, 1992 is the starting point for studies on HIM in Brazil. During the total of 32 years (Figure 3A), there was an average of 4.9 studies per year. The number of studies increased from 1992 to 2020 and decreased thereafter. The most significant number of studies was published in 2020 (10 documents), followed by 2015, and 2021 (9); 2009, 2011, 2012, and 2017 (8), and in each of the other years, there were ≤ 7. No studies were conducted between 1993 and 1995. Fifty percent of the studies were published from 2013 to 2024, and 70% from 2009 to 2024.

### 6.2 Types of Food Groups Analyzed Over the Years

The studies were categorized according to the food group, as animal or plant origin. In some of the documents, more than one food group was investigated, e.g., eight in Batista (2007), three in Molognoni et al. (2018), and two food groups in 12 documents (Silva 2000; Sabaini 2009; Fraga 2010; Rigueira 2010; Ubaldo et al. 2015; Montanha 2016; Mota et al. 2018; Cavalcante et al. 2019; Dala‐Paula, Starling, et al. 2021; Rocha 2021; Basilio et al. 2022; Dala‐Paula et al. 2024). Half of the documents concerned foods of animal origin, while the other half concerned foods of plant origin.

The first studies on foods of animal origin were published in 1992 (Mendonça 1992; Soares 1992). They reported HIM in fresh, canned, or processed fish. These studies addressed worldwide concerns regarding HIM intoxication (Taylor 1985). In 1996, studies began on cheese (Vale 1996; Vale and Gloria 1997, 1998), the second type of food associated with HIM intoxication (EFSA 2011, 2017; Ferrante and Mercogliano 2023). Next, a wider range of foods was investigated. The publication/time trend for food of animal origin (Figure 3B) was like that of the total. A significant increase in the number of documents was observed up to 2018, followed by 2013 and 2016, and ≤ 5 for the other years. Studies on fish and dairy products were carried out over the entire period. Documents on meat and dry‐fermented sausages were published in two different periods, 2000 to 2008 and 2013 to 2023. Publications about eggs were available from 2009 to 2016. Documents on fish, fish products, milk, and cheese were the most prevalent (33.3%), followed by meat and dry‐fermented sausages (28.7%). In contrast, the documents on eggs represented only 5.7% of the studies. Studies on honey were first reported in 2023.

Alcoholic beverages (beer) were the first food of plant origin investigated (Gloria and Izquierdo‐Pulido 1999), five years after food of animal origin (Figure 3C). The number of documents per year was higher in 2007, followed by 2022, 2021, 2011, and 2020; the remainder were ≤ 5 per year. Documents on HIM in alcoholic beverages and fruits were prevalent (16.5%), followed by vegetables (10.6%), pulse and derivatives, cocoa and chocolate (8.2%), fruit derivatives, Coffee (7.1%), and the others were < 6%. Publications on alcoholic beverages prevailed from 1999 to 2017, fruit derivatives from 2007 to 2022, fruits from 2004 to 2024, vegetables from 2001 to 2024, cocoa and chocolate from 2017 to 2023, and mushrooms in 2020.

When considering the purpose of the studies, 85.8% reported the contents of amines in food for health purposes, including both beneficial and adverse effects. They investigated the influence of species, agricultural practices, production, degree of ripening, and processing on HIM content. The other studies (12.3%) focused on the development and optimization of methods of analysis. In contrast, in a few recent studies (1.9%), starting in 2020, the aim was to determine the in vitro digestion of amines (Reis, Dala‐Paula, et al. 2020; Dala‐Paula, Deus, et al. 2021; Silva et al. 2023).

### 6.3 Methods Used for Histamine Analysis

The most widely used method for HIM analysis in the documents involved extraction from the food using trichloroacetic acid—TCA (71.8%), separation by high‐performance liquid chromatography—HPLC (92.3%), post‐column derivatization with *o*‐phthalaldehyde—OPA (67.9%), and fluorescence detection (96.2%). HIM extraction from the food is vital to remove interferences and impurities and ensure the analytical results' validity (Koo and Lim 2023). Acidic solvents are paramount as they allow protein precipitation and stabilization of the amine (Tsiasioti et al. 2023). Because amines do not have chromophores, HIM must be derivatized for quantification (Önal et al. 2013). In addition, derivatization enhances the analytical techniques' sensitivity, selectivity, and compatibility (Koo and Lim 2023; Tsiasioti et al. 2023). OPA is a versatile derivatization reagent, commercially available reasonably cheaply, and reacts rapidly under mild conditions (Tsiasioti et al. 2023). Online post‐column derivatization provides a more stable derivative, higher recovery, reproducibility, and ease and speed of analysis (Tırıs et al. 2023; Tsiasioti et al. 2023). Fluorescence detection offers high sensitivity and selectivity for detecting HIM in food (Tırıs et al. 2023).

In most documents (84.6%), six or more amines and HIM were analyzed simultaneously. The single analysis of HIM was reported in only 10.9% of the documents, mainly in the 90's. At that time, the most widely used method was the AOAC official HIM method (AOAC 1980).

Even though different methods were used in the HIM analysis, they were validated and demonstrated to be reliable, accurate, and precise for analyzing HIM in food. The limit of quantification (LOQ) was provided in several documents. They varied from 0.02 to 4.0 mg/kg. The lowest LOQ was evidenced in fish by the AOAC official method—0.02 mg/kg (Soares and Gloria 1994; Soares et al. 1998). This higher sensitivity is probably due to purification steps, derivatization with OPA, and FL detection. When considering the most widely used method (HPLC, post‐column derivatization with OPA and FL detection), the LOQ was affected by the food matrix. The lowest LOQ (0.1 mg/kg) was observed for fish and meat (Evangelista et al. 2016; Custódio et al. 2018). It was followed by 0.2 mg/L for fermented beverages and sauces (Gloria and Izquierdo‐Pulido 1999; Manfroi et al. 2009; Guidi and Gloria 2012), 0.8 mg/kg for fruits and vegetables (Santiago‐Silva et al. 2011; Dala‐Paula, Starling, et al. 2021), and 1.0 mg/kg for eggs (Oliveira et al. 2009; Figueiredo et al. 2013). The higher LOQs (3.0 and 4.0 mg/kg) were reported for cheese and dry‐fermented sausages, respectively (Vale and Gloria 1998; Moreira et al. 2018; Braga 2023), which would not be a concern since these products are usually rich in HIM. These LOQs are similar to or even lower than those reported in the literature (Ordóñez et al. 2016; Tırıs et al. 2023; Tsiasioti et al. 2023). Based on these results, it is safe to assume that, generally, the detectable levels can be considered ≥ 1 mg/kg, except for dry‐fermented sausage and cheese.

## 7 Histamine Levels in Brazilian Food

HIM levels in food were available for 343 different types of food. For food of animal origin, there were 156 types of food and 9151 samples with an average of 58.7 samples/food, whereas there were 187 types and 2310 samples, with an average of 12.4 samples per type of food of plant origin. The HIM levels are displayed for individual foods of animal (Table 3) and plant origins (Table 4). The results were reported as mean, minimum, and maximum values, depending on the information in the respective documents.

**TABLE 3:** Mean levels and range (minimum and maximum) of histamine (mg/kg or mg/L) in Brazilian food of animal origin and number of samples analyzed.

Foods	Sample	Level (mg/kg or mg/L)			References
		Mean	Min	Max	
*Milk & derivatives*					
Sheep					
Milk, sheep, freeze‐dried	9	0.13	0.11	0.14	Montanha (2016)
Cheese, sheep, fresh	3	nd	—	—	Montanha (2016)
Cheese, sheep, aged	6	nd	—	—	Montanha (2016)
Goat					
Milk, fermented	3	53.9	—	—	Costa et al. (2015)
Cow					
Milk, raw, cow	6	nd	—	—	Gloria et al. (2011)
Milk, raw, cow	13	nd	—	—	Rigueira et al. (2011)
Milk, raw, cow	9	nd	—	—	Ubaldo et al. (2015)
Milk, UHT	10	nd	—	—	Almeida et al. (2003)
Milk, dried, whole	10	nd	—	—	Batista (2007)
Milk, dried, reconstituted	4	nd	—	—	Santos et al. (2003)
Milk, pasteurized	3	0.50	nd	0.93	Santos et al. (2020)
Milk, fermented	10	nd	—	—	Ciríaco (2018)
Milk, fermented	15	nd	—	—	Ciríaco et al. (2019)
Milk, fermented	3	18.0	—	—	Costa et al. (2015)
Beverage, dairy	14	nd	—	—	Rigueira et al. (2011)
Whey, Cheese	16	0.05	nd	0.20	Siqueira (2000)
*Non‐aged cheese*					
Cheese, artisanal, Serra Geral, fresh	24	63.8	63.2	64.4	Caldeira et al. (2024)
Cheese, Minas, Frescal, fresh	10	6.90	—	—	Cunha et al. (2012)
Cheese, Minas, Padrão	16	3.30	—	—	Vale and Gloria (1998)
Cheese, Minas, Padrão	3	nd	—	—	Santos et al. (2017)
Cheese, Mozzarella	10	2.22	nd	22.2	Rigueira (2010)
Cheese, Mozzarella	9	nd	—	—	Rigueira (2010)
Cheese, Mozzarella	13	15.7	—	—	Vale and Gloria (1998)
Cheese, Mozzarella	18	nd	—	—	Moreira (2018)
Cheese, Mozzarella	10	71.7	—	—	Cunha et al. (2012)
Cheese, Mozzarella	18	nd	—	—	Ubaldo et al. (2015)
Cheese, Requeijão cream	14	0.20	—	—	Vale (1996)
Cheese, Requeijão bar	7	1.40	—	—	Vale (1996)
*Aged cheese*					
Cheese, artisanal, Serra Geral, aged	72	35.1	21.5	46.4	Rocha (2021)
Cheese, artisanal, Serro, aged	24	132	19.2	423	Araújo (2013b)
Cheese, artisanal, Serro, aged	10	16.2	6.34	33.9	Cardoso (2011)
Cheese, Gorgonzola	3	13.5	—	—	Vale and Gloria (1998)
Cheese, Gorgonzola	18	4.00	nd	24.0	Moreira et al. (2018)
Cheese, Gorgonzola	3	nd	—	—	Santos et al. (2017)
Cheese, Gorgonzola	6	24.3	nd	65.5	Santos et al. (2020)
Cheese, Gouda	9	28.4	—	—	Vale and Gloria (1998)
Cheese, Gouda	10	79.1	—	—	Cunha et al. (2012)
Cheese, Gruyere	3	nd	—	—	Santos et al. (2017)
Cheese, Moleson	3	nd	—	—	Santos et al. (2017)
Cheese, Parmesan	6	2.10	—	—	Vale and Gloria (1998)
Cheese, Parmesan	6	107	—	—	Vale and Gloria (1997)
Cheese, Parmesan	18	nd	—	—	Moreira (2018)
Cheese, Prato	15	16.0	—	—	Vale and Gloria (1998)
Cheese, Prato	10	14.8	—	—	Cunha et al. (2012)
Cheese, Prato	3	nd	—	—	Santos et al. (2017)
Cheese, Provolone	15	10.4	—	—	Vale and Gloria (1998)
Cheese, Reblochon	3	nd	—	—	Santos et al. (2017)
Cheese, Sbrinz	3	nd	—	—	Santos et al. (2017)
Cheese, Tilsit	3	16.1	—	—	Vale and Gloria (1998)
*Grated cheese*					
Cheese, Grated	9	104	2.20	257	Custódio (2006)
Cheese, Grated	4	28.8	—	—	Vale and Gloria (1998)
Cheese, Grated	4	104	19.8	1154	Pimentel (2001)
Cheese, Grated, Parmesan	30	108	1.00	521	Pimentel (2001)
Cheese, Grated, Parmesan	21	213	2.10	783	Custódio et al. (2007)
Cheese, Grated, Parmesan	8	37.1	—	—	Vale and Gloria (1998)
Cheese, Grated, Parmesan	9	124	9.00	251	Pimentel (2001)
Cheese, Grated, Parmesan	9	103	2.20	257	Custódio (2006)
Cheese, Grated, Parmesan	34	49.1	nd	215	Moreira and Gloria (2017)
*Meat & derivatives*					
Beef					
Liver	16	0.12	0.02	0.22	Custódio et al. (2016)
Liver, grilled	5	0.04	—	—	Custódio et al. (2016)
Liver, stored	44	0.05	0.01	0.09	Custódio et al. (2016)
Meat, ground	14	nd	—	—	Batista (2007)
Meat, Angus, fresh	6	2.90	2.70	3.10	Filgueras (2007)
Meat, Zebuin, fresh	6	2.40	2.00	2.80	Filgueras (2007)
Meat, Zebuin, aged	24	1.98	1.30	2.80	Filgueras (2007)
Meat, Angus, aged	24	2.21	1.09	2.70	Filgueras (2007)
Meat, industrial	4	2.20	—	—	Silva (2000)
Corned beef	20	nd	—	—	Molognoni et al. (2018)
Jerked beef	3	nd	—	—	Molognoni et al. (2018)
Chicken					
Breast	8	nd	—	—	Silva and Gloria (2002)
Breast	84	5.01	3.35	6.06	Lázaro et al. (2014)
Breast	160	0.88	0.60	1.40	Assis et al. (2015)
Breast	12	2.94	2.91	2.97	Assis et al. (2016)
Breast, aged	39	0.03	nd	0.10	A. P. S. Moreira (2005)
Breast, aged	30	nd	—	—	Moreira et al. (2008)
Breast, aged, cooked	9	0.16	nd	0.47	Moreira et al. (2008)
Breast, cooked	10	nd	—	—	Batista (2007)
Fillet, ready‐to‐eat frozen	90	0.08	nd	0.14	Baptista et al. (2014)
Skin	4	0.30	—	—	Silva (2000)
Thigh	12	0.40	nd	0.70	Silva and Gloria (2002)
Meat, mechanically separated	4	2.30	—	—	Silva (2000)
Hamburger	10	0.20	—	—	Silva and Gloria (2002)
Meatball	10	nd	—	—	Silva and Gloria (2002)
Mortadella	10	1.50	—	—	Silva and Gloria (2002)
Nugget	10	nd	—	—	Silva and Gloria (2002)
Salsichão	10	0.90	—	—	Silva and Gloria (2002)
Sausage	10	13.5	—	—	Silva and Gloria (2002)
Pork					
Bacon	2	nd	—	—	Molognoni et al. (2018)
Leg	42	nd	—	—	Custódio et al. (2018)
Loin	42	nd	—	—	Custódio et al. (2018)
Pork jowl	4	0.20	—	—	Silva (2000)
Alligator					
Meat, frozen	35	1.02	0.04	1.60	Neto (2008)
Meat, glazed, frozen	35	0.66	0.04	1.00	Neto (2008)
Tail fillet	10	1.52	1.44	1.59	Morais (2013)
Derivatives					
Calabresa, raw sausage	5	nd	—	—	Molognoni et al. (2018)
Ham	3	nd	—	—	Molognoni et al. (2018)
Mortadella	69	1.31	0.20	2.51	Alves (2017)
Pepperoni	3	nd	—	—	Molognoni et al. (2018)
Salame	3	nd	—	—	Molognoni et al. (2018)
Salame, cooked	3	48.1	—	—	Pena (2006)
Dried‐cured pork loin	21	131	117	145	Mutz et al. (2021)
Sausage, dry‐fermented, Brianza	6	44.1	—	—	Caccioppoli (2002)
Sausage, dry‐fermented, Friolano	6	42.4	—	—	Caccioppoli (2002)
Sausage, dry‐fermented, Hamburgues	18	5.37	nd	16.1	Caccioppoli (2002)
Sausage, dry‐fermented, Hamburgues	6	336	nd	1343	Braga (2023)
Sausage, dry‐fermented, Hamburgues mini	6	nd	—	—	Braga (2023)
Sausage, dry‐fermented, Italiano	6	13.8	—	—	Caccioppoli et al. (2006)
Sausage, dry‐fermented, Italiano	36	33.4	0.90	121	Caccioppoli (2002)
Sausage, dry‐fermented, Italiano	10	138	nd	500	Roselino et al. (2020)
Sausage, dry‐fermented, Italiano	6	90.7	24.5	170	Santos et al. (2015)
Sausage, dry‐fermented, Italiano	3	146	—	—	Pena (2006)
Sausage, dry‐fermented, Italiano	30	102	58.7	143	Caccioppoli (2002)
Sausage, dry‐fermented, Italiano	9	44.1	nd	186	Braga (2023)
Sausage, dry‐fermented, Italiano gourmet	9	515	nd	1301	Braga (2023)
Sausage, dry‐fermented, Italiano gourmet	6	598	270	799	Braga (2023)
Sausage, dry‐fermented, Milano	37	36.1	nd	195	Braga (2023)
Sausage, dry‐fermented, Milano	3	183	—	—	Braga (2023)
Sausage, dry‐fermented, Milano	3	37.2	—	—	Braga (2023)
Sausage, dry‐fermented, Salaminho	18	1.17	0.10	3.30	Caccioppoli (2002)
Sausage, dry‐fermented, Salaminho	41	15.4	nd	130	Braga (2023)
Sausage, dry‐fermented, Salaminho	6	nd	—	—	Braga (2023)
Sausage, dry‐fermented, Serrano	4	20.8	1.16	41.4	Braga (2023)
Fish & derivatives					
*Non‐susceptible to histamine formation*					
Abrotea, *Urophycis brasiliensis*, Phycidae	8	nd	—	—	Soares et al. (1998)
Bagre, *Clarias gariepinus* , Ariidae, pisciculture	8	nd	—	—	Brandão (1996)
Cação, Carcharhinidae Squalidae, frozen	16	0.60	nd	1.30	Soares et al. (1998)
Cambeua, *Notarius grandicassis*, Ariidae, salted	20	4.40	—	—	Battagin (2017)
Carpa, *Cyprinus carpio* , Cyprinidae, pisciculture	8	nd	—	—	Brandão (1996)
Castanha, *Umbrina* sp., Sciaenidae, frozen	16	0.60	nd	3.70	Soares et al. (1998)
Congro Genypterus biacodes, Congridae, frozen	8	2.4	nd	5.70	Soares et al. (1998)
Corvina, *Whitemouth croak*er, Sciaenidae	8	4.20	—	—	Soares et al. (1998)
Curimatã, *Prochilodus nigricans* , Prochilodontidae	24	nd	—	—	Soares et al. (2021)
Jaraqui, *Semaprochilodus insignis* , Prochilodontidae	24	nd	—	—	Soares et al. (2021)
Linguado, *Paralichthys* sp., *Pleuronectes* sp., Paralichthyidae, frozen	8	0.0	nd	0.20	Soares et al. (1998)
Merluza *Merlucius hubbsi*, Merlucciidae, frozen	16	1.90	nd	5.20	Soares et al. (1998)
Namorado, *Pseudopercis numida* , Pinguipedidae, frozen	8	5.00	nd	13.0	Soares et al. (1998)
Pacu, *Piaractus mesopotamicus* , Serrasalmidae, pisciculture	8	nd	—	—	Brandão (1996)
Pacu, *Mylossoma duriventre* , Serrasalmidae	24	nd	—	—	Soares et al. (2021)
Pescada, *Macrodon oncylodon*, Sciaenidae, frozen	20	0.50	nd	2.40	Soares et al. (1998)
Pescada Go, *Macrodon ancylon*, Sciaenidae, salted	20	2.73	—	—	Battagin (2017)
Pescadinha, *Cynoscion stnatus*, Merlucciidae	12	0.40	nd	3.50	Soares et al. (1998)
Piramutaba, *Branchyplatystoma vaillantii*, Pimelodidae, salted	20	4.13	—	—	Battagin (2017)
Rainbow trout, *Oncorhynchus mykiss* , Salmonidae	3	nd	—	—	Evangelista et al. (2016)
Robalo, *Centropomus parallelus*, Centropomidae	12	37.4	5.69	68.8	Baliero (2010)
Tambaqui *Colossoma macropomum* , Serrasalmidae, pisciculture	8	nd	—	—	Brandão (1996)
Tainha, *Mugil platanus*, Mugilidae	2	106	78.4	134	Andrade et al. (2014)
Tilapia, *Sarotherodon hornorum*, Cichlidae, pisciculture	8	nd	—	—	Brandão (1996)
Tilapia, *Oreochromis niloticus* , Cichlidae	15	nd	—	—	Evangelista et al. (2016)
Tilapia, *O. niloticus* , Cichlidae, paste	5	nd	—	—	Matiucci et al. (2023)
*Susceptible to histamine formation*					
Carangidae					
Chicharro, grated, canned	6	2.60	0.90	3.50	Soares and Gloria (1994)
Timbira, *Oligoplites palometo*	40	0.10	nd	0.60	Araújo (2013a)
Xareu, *Caranx ignobilis*	50	0.26	nd	0.90	Araújo (2013a)
Clupeidae					
Sardinha, *Cetengraulis edentulous*	9	—	nd	1.8	Andrade et al. (2012)
Sardinha, *Triportheus angulatus*	24	nd	—	—	Soares et al. (2021)
Sardinha, *Sardinella brasiliensis* , fresh	12	403	226	580	Lira et al. (2020)
Sardinha, *Sardinella brasiliensis* , fresh	10	—	nd	1.8	Andrade et al. (2012)
Sardinha, *Sardinella brasiliensis* , frozen	12	463	343	584	Lira et al. (2020)
Sardinha, *Sardinella brasiliensis* , red meat	24	nd	—	—	Soares et al. (2005)
Sardinha *Sardinella brasiliensis* , white meat	24	nd	—	—	Soares et al. (2005)
Sardine, canned in oil	10	3.00	2.00	6.00	Mendonça (1992)
Sardine, canned in oil	9	nd	—	—	Molognoni et al. (2018)
Sardine, canned in oil	47	6.40	—	—	Soares and Gloria (1994)
Sardine, fillet, canned in oil	8	6.00	—	—	Soares and Gloria (1994)
Sardine, no skin & spine, canned in oil	15	5.80	—	—	Soares and Gloria (1994)
Sardinha, fillet, canned in oil with pepper	10	17.5	—	—	Soares and Gloria (1994)
Sardinha, fillet, canned in oil with onion & bay leaves	9	15.5	—	—	Soares and Gloria (1994)
Sardinha, fillet, canned in oil with pickles	6	10.8	—	—	Soares and Gloria (1994)
Sardinha, fillet, no spine, canned, tomato sauce	8	5.90	—	—	Soares and Gloria (1994)
Sardinha, fillet, canned, tomato sauce	14	8.60	—	—	Soares and Gloria (1994)
Sardinha, paste	6	19.8	—	—	Soares and Gloria (1994)
Sardinha, fermented	9	6.40	1.80	11.0	Pombo et al. (2009)
Engraulidae					
Anchovy, canned	3	nd	—	—	Molognoni et al. (2018)
Anchovy, canned	6	nd	—	—	Soares and Gloria (1994)
Scombridae					
Bonito, Scombridae, grated, canned in oil	2	56.0	—	—	Mendonça (1992)
Bonito, Scombridae, grated, canned in oil	4	15.2	—	—	Soares and Gloria (1994)
Bonito, Scombridae, solid, canned in oil	6	2.9	1.10	5.40	Mendonça (1992)
Cavala, *Scomberomorus cavalla*	12	43.2	42.5	43.8	Baliero (2010)
Cavala, Scombridae	30	4.03	nd	11.9	Araújo (2013a)
Sarda, *Scomber scombrus*	30	0.10	nd	0.40	Araújo (2013a)
Serra *Scomberomorus brasiliensis*	40	nd	—	—	Araújo (2013a)
Tuna	11	nd	—	—	Evangelista et al. (2016)
Tuna	180	5.77	nd	6.90	Oliveira et al. (2012)
Tuna	31	69.3	nd	649	Evangelista (2015)
Tuna	180	nd	—	—	Oliveira et al. (2012)
Tuna	4	2.00	1.62	2.54	Tavares (2012)
Tuna albacore, *Thunnus obesus*	24	nd	—	—	Oliveira et al. (2012)
Tuna in brine, *Thunnus* spp.	3	nd	—	—	Olivo (2013)
Tuna in ice, *Thunnus* spp.	3	17.5	nd	38.4	Olivo (2013)
Tuna, *Katsuwonus pelamis* , frozen	3	nd	—	—	Barbosa et al. (2017)
Tuna, *Katsuwonus pelamis* , thawed	3	nd	—	—	Barbosa et al. (2017)
Tuna	117	nd	—	—	Evangelista et al. (2016)
Tuna	3	52.1	—	—	Vitali et al. (2013)
Tuna	429	0.76	nd	4.64	Silva et al. (2011)
Tuna, bandolim, *Thunnus obesus*	24	nd	—	—	Oliveira (2009)
Tuna, laje, *Thunnus albacares*	24	nd	—	—	Oliveira (2009)
Tuna, *Katsuwonus pelamis* , solid, cooked	3	3.57	nd	5.8	Barbosa et al. (2017)
Tuna, *Katsuwonus pelamis* , grated, cooked	3	3.53	nd	10.6	Barbosa et al. (2017)
Tuna, solid, canned in oil	6	2.73	2.00	5.00	Mendonça (1992)
Tuna, canned	3	20	—	—	Oliveira et al. (2023)
Tuna, solid, canned in oil	2	nd	—	—	Schulz (2009)
Tuna, solid, canned in oil	9	1.32	nd	11.1	Silva et al. (2011)
Tuna, solid, canned in oil	33	8.90	—	—	Soares and Gloria (1994)
Tuna, grated, canned in oil	12	28.0	nd	56.0	Evangelista et al. (2016)
Tuna, solid, canned in water & salt	2	nd	—	—	Schulz (2009)
Tuna, solid, canned in water & salt	3	0.82	—	—	Vitali et al. (2013)
Tuna, solid, light, canned	3	8.96	—	—	Vitali et al. (2013)
Tuna, solid, canned	7	11.1	—	—	Soares and Gloria (1994)
Tuna, solid, canned in water & salt	44	40.7	nd	81.4	Evangelista et al. (2016)
Tuna, solid, canned in water & salt	9	0.16	nd	1.01	Silva et al. (2011)
Tuna, grated, canned in oil	12	9.57	nd	56.2	Silva et al. (2011)
Tuna, grated, canned in oil	12	28.0	nd	56.0	Evangelista et al. (2016)
Tuna, grated, canned in oil	10	1.64	1.00	3.00	Mendonça (1992)
Tuna, grated, canned in oil	2	nd	—	—	Schulz (2009)
Tuna, grated, canned in oil	6	18.6	—	—	Soares and Gloria (1994)
Tuna, grated, canned in water & salt	3	nd	—	—	Schulz (2009)
Tuna, grated, canned in water & salt	3	1.08	—	—	Vitali et al. (2013)
Tuna, grated, canned in water & salt	12	1.03	nd	2.06	Evangelista et al. (2016)
Tuna, grated, canned in water & salt	12	0.36	nd	2.06	Silva et al. (2011)
Tuna, *Katsuwonus pelamis* , grated, canned in water and salt	3	nd	—	—	Barbosa et al. (2017)
Tuna, seasoned, canned	2	2.2	—	—	Mendonça (1992)
Shellfish (crustaceans)					
Crab	16	222	53	537	Baliero (2010)
Shrimp	16	1.40	0.40	2.10	Soares (1992)
Fish dishes					
Sashimi, salmon	10	91.6	23.0	163	Cordeiro et al. (2020)
Sashimi, salmon	14	85.5	3.60	167	Rodrigues et al. (2012)
Sushi, tuna	3	6.39	—	—	Vitali et al. (2013)
Sushi, salmon	18	0.60	—	—	Rodrigues et al. (2012)
*Egg & derivatives*					
Egg white	40	3.06	0.31	13.3	Assis et al. (2016)
Egg white	2400	nd	—	—	Figueiredo et al. (2013, 2014)
Egg white	120	nd	—	—	Oliveira (2006); Oliveira et al. (2009)
Egg yolk	40	0.21	nd	0.56	Assis et al. (2016)
Egg yolk	120	nd	—	—	Oliveira (2006); Oliveira et al. (2009)
Egg yolk	2400	nd	—	—	Figueiredo et al. (2013, 2014)
Boiled egg	7	nd	—	—	Batista (2007)
Liquid pasteurized egg	24	nd	—	—	Rêgo et al. (2014)
*Honey*					
Honey, São Paulo	64	24.1	2.83	181	Lima et al. (2023)
Honey, Santa Catarina	50	16.1	1.58	37.7	Lima et al. (2023)

*Note:* nd, not detected (below detectable level ≤ 1.0 mg/kg; except for cheese and dry‐fermented sausage, ≤ 3.0 and 4.0 mg/kg, respectively). (—) not available.

**TABLE 4:** Mean levels and range (minimum and maximum) of histamine (mg/kg or mg/L) in Brazilian food of plant origin and number of samples analyzed.

Foods	Sample	Levels (mg/kg or mg/L)			References
		Mean	Min	Max	
*Fruits*					
Açai ( *Euterpe oleracea* M.)	5	nd	—	—	Faria (2011)
Acerola ( *Malpighia glabra* L.)	3	nd	—	—	Dala‐Paula et al. (2024)
Apple ( *Malus domestica* )	7	nd	—	—	Santiago‐Silva (2004)
Avocado ( *Persea americana* M.)	5	nd	—	—	Faria (2011)
Banana ( *Musa sapientum* )	12	nd	—	—	Adão and Gloria (2005)
Bergamot ( *Citrus bergamia* ) pulp+juice	3	28.4	—	—	Belin et al. (2024)
Custard apple ( *Annona squamosa* )	3	nd	—	—	Dala‐Paula et al. (2024)
Genipap ( *Genipa americana* L.)	5	nd	—	—	Faria (2011)
Grape, BRS Carmen	10	nd	—	—	Mota et al. (2018)
Grape, Niagara	16	nd	—	—	Sabaini (2009)
Grape, Niagara pink ( *Vitis labrusca* L. × *V. vinífera* L.)	9	1.32	0.27	2.25	Gomes et al. (2021)
Grape, Ruby	16	nd	—	—	Sabaini (2009)
Grape, Syrah ( *V. vinifera* L.)	16	nd	—	—	Sabaini (2009)
Grape, Syrah ( *V. vinifera* L.)	40	nd	—	—	Fraga (2010)
Guava, red ( *Psidium guajava* L.)	7	nd	—	—	Santiago‐Silva et al. (2011)
Jabuticaba ( *Plinia cauliflora* )	3	nd	—	—	Dala‐Paula et al. (2024)
June plum ( *Spondias dulcis* ) P.	5	nd	—	—	Faria (2011)
Khaki ( *Diospyros kaki* L.)	5	nd	—	—	Faria (2011)
Lemon ( *Citrus limon* L.)	14	nd	—	—	Batista (2007)
Marolo (*Annona crassifora*)	3	nd	—	—	Ávila et al. (2024)
Mango ( *Mangifera indica* L.) Tommy	7	nd	—	—	Santiago‐Silva et al. (2011)
Melon ( *Cucumis melo* )	7	0.5	—	—	Santiago‐Silva (2004)
Orange ( *Citrus sinensis* L.)	14	0.1	—	—	Batista (2007)
Papaya ( *Carica papaya* L.)	7	nd	—	—	Santiago‐Silva et al. (2011)
Passion fruit (*Passiflora tenuifila* Kilip)	20	nd	—	—	Santos et al. (2021)
Passion fruit ( *P. alata* Dryand)	3	nd	—	—	Bomtempo et al. (2016)
Passion fruit ( *P. alata* Dryand)	14	nd	—	—	Santiago‐Silva et al. (2011)
Passion fruit ( *P. edulis* )	3	nd	—	—	Bomtempo et al. (2016)
Passion fruit ( *P. nitida* )	3	nd	—	—	Bomtempo et al. (2016)
Passion fruit ( *P. setacea* )	21	nd	—	—	Bomtempo et al. (2016)
Passion fruit ( *P. setacea* )	3	nd	—	—	Sanchez et al. (2020)
Passion fruit (*P. tenuifila* Kilip)	4	nd	—	—	Bomtempo et al. (2016)
Peach ( *Prunus persica* )	7	2.8	—	—	Santiago‐Silva (2004)
Pear ( *Pyrus communis* L.)	5	nd	—	—	Faria (2011)
Pequi ( *Caryocar brasiliense* C.)	5	nd	—	—	Faria (2011)
Pineapple ( *Ananas comosus* L.)	7	nd	—	—	Santiago‐Silva et al. (2011)
Seriguela ( *Spondias purpurea* L.)	3	nd	—	—	Dala‐Paula et al. (2024)
Soursop ( *Annona muricata* ) L.	5	nd	—	—	Faria (2011)
Star fruit *Averrhoa carambola* L.	5	nd	—	—	Faria (2011)
Strawberry (*Fragaria × ananassa*)	7	nd	—	—	Santiago‐Silva (2004)
Umbu ( *Spondias tuberosa* A. K.)	5	nd	—	—	Faria (2011)
Watermelon ( *Citrullus lanatus* )	7	0.7	—	—	Santiago‐Silva (2004)
*Fruit derivatives*					
Orange, juice	21	0.10	0.03	0.26	Vieira et al. (2007)
Orange, juice	3	nd			Vieira et al. (2010)
Orange, soft drink	35	0.00	nd	0.03	Vieira et al. (2007)
Grape, juice, Niagara rosada	3	0.09	—	—	Monteiro (2020)
Grape, juice, Máximo	3	1.00	—	—	Monteiro (2020)
Grape, juice, Bordo	3	3.47	—	—	Monteiro (2020)
Grape juice, Bordo	10	nd	—	—	Mota et al. (2018)
Grape juice, Violeta	3	1.30	—	—	Monteiro (2020)
Grape juice, Violeta, BRS	10	nd	—	—	Mota et al. (2018)
Grape juice, Isabel, precoce	10	nd	—	—	Mota et al. (2018)
Grape juice, *Vitis labrusca*	14	nd	—	—	Nassur et al. (2014)
Grape must, violeta	3	0.04	—	—	Basilio et al. (2022)
Grape must, cora	3	0.04	—	—	Basilio et al. (2022)
Grape must, bordo	3	0.17	—	—	Basilio et al. (2022)
Grape, Syrah ( *Vitis vinifera* L.) seed	8	nd	—	—	Fraga (2010)
Grape, Syrah ( *V. vinifera* L.) peel	8	nd	—	—	Fraga (2010)
Grape, peel flour	12	0.12	0.08	0.60	Monteiro et al. (2021)
*Vegetables*					
Almeirão roxo ( *Lactuca canadensis* L.)	3	nd	—	—	Dala‐Paula et al. (2024)
Beet ( *Beta vulgaris* L.)	14	0.50	—	—	Batista (2007)
Beet, cooked ( *B. vulgaris* L.)	14	1.10	—	—	Batista (2007)
Broccoli ( *Brassica oleracea var. italica* )	10	nd	—	—	Dala‐Paula, Starling, et al. (2021)
Capers ( *Capparis spinosa* L.)	10	nd	—	—	Dala‐Paula, Starling, et al. (2021)
Carrot ( *Daucus carota* L.)	14	nd	—	—	Batista (2007)
Carrot, cooked ( *D. carota* L.)	14	nd	—	—	Batista (2007)
Cassava ( *Manihot esculenta* Crantz)	5	nd	—	—	Dala‐Paula, Starling, et al. (2021)
Cauliflower ( *Brassica oleracea* botrytis L.)	10	nd	—	—	Dala‐Paula, Starling, et al. (2021)
Cauliflower ( *B. oleracea* botrytis L.), Verdi, raw	3	nd	—	—	Diamante et al. (2019)
Cauliflower ( *B. oleracea* botrytis L.), Verdi, cooked	6	0.01	—	—	Diamante et al. (2019)
Cauliflower ( *B. oleracea* botrytis L.), Verdi, steamed	6	0.01	—	—	Diamante et al. (2019)
Cauliflower ( *B. oleracea* botrytis L.), Verdi, microwaved	6	0.03	—	—	Diamante et al. (2019)
Cauliflower ( *B. oleracea* botrytis L.), Forata, raw	3	nd	—	—	Diamante et al. (2019)
Cauliflower ( *B. oleracea* botrytis L.), Forata, cooked	6	0.04	0.03	0.04	Diamante et al. (2019)
Cauliflower ( *B. oleracea* botrytis L.), Forata, steamed	6	0.04	0.03	0.04	Diamante et al. (2019)
Cauliflower ( *B. oleracea* botrytis L.), Forata, microwaved	6	0.04	0.03	0.04	Diamante et al. (2019)
Cauliflower ( *B. oleracea* botrytis L.), Cheddar, raw	3	0.03	—	—	Diamante et al. (2019)
Cauliflower ( *B. oleracea* botrytis L.), Cheddar, cooked	6	0.03	0.02	0.03	Diamante et al. (2019)
Cauliflower ( *B. oleracea* botrytis L.), Cheddar, steamed	6	0.01	—	—	Diamante et al. (2019)
Cauliflower ( *B. oleracea* botrytis L.), Cheddar, microwaved	6	0.02	—	—	Diamante et al. (2019)
Cauliflower ( *B. oleracea* botrytis L.), Graffiti, raw	3	0.01	—	—	Diamante et al. (2019)
Cauliflower ( *B. oleracea* botrytis L.), Graffiti, cooked	6	0.01	—	—	Diamante et al. (2019)
Cauliflower ( *B. oleracea* botrytis L.), Graffiti, steamed	6	0.01	—	—	Diamante et al. (2019)
Cauliflower ( *B. oleracea* botrytis L.), Graffiti, microwaved	6	0.01	—	—	Diamante et al. (2019)
Chayote ( *Sechium edule* ), cooked	14	nd	—	—	Batista (2007)
Collard greens ( *Brassica oleracea* acephala)	10	nd	—	—	Vieira (2003)
Eggplant ( *Solanum melongena* L.)	10	83.2	36.9	125	Dala‐Paula, Starling, et al. (2021)
Eggplant ( *S. melongena* L.), peel	10	101	48.5	161	Dala‐Paula, Starling, et al. (2021)
Eggplant ( *S. melongena* L.), pulp	10	2.9	1.80	4.60	Dala‐Paula, Starling, et al. (2021)
Eggplant ( *S. melongena* L.), core	10	82.0	75.2	93.6	Dala‐Paula, Starling, et al. (2021)
Hearts of palm ( *Euterpe oleracea* Mart.)	10	nd	—	—	Dala‐Paula, Starling, et al. (2021)
Kale ( *Brassica oleracea* L.)	3	nd	—	—	Dala‐Paula et al. (2024)
Lettuce ( *Lactuca sativa* L.)	42	nd	—	—	Dala‐Paula (2012)
Lettuce, American ( *L. sativa* cv Lucy Brown)	9	nd	—	—	Coelho (2001)
Lettuce, American ( *L. sativa* cv Lucy Brown)	36	nd	—	—	Coelho et al. (2005)
Lettuce, American, minimally processed ( *L. sativa* cv Lucy Brown)	27	nd	—	—	Coelho (2001)
Onion ( *Allium cepa* L)	10	nd	—	—	Batista (2007)
Onion ( *Allium fistulosum* ), green	10	nd	—	—	Dala‐Paula, Starling, et al. (2021)
Ora‐pro‐nobis ( *Pereskia aculeata* Mill.)	3	1.25	1.08	1.44	Dala‐Paula et al. (2024)
Parsley ( *Petroselinum hortense* )	10	nd	—	—	Dala‐Paula, Starling, et al. (2021)
Potato ( *Solanum tuberosum* L.), cooked	14	nd	—	—	Batista (2007)
Potato, sweet	3	0.60	—	—	Basilio et al. (2022)
Pumpkin ( *Cucurbita maxima* )	14	5.10	—	—	Batista (2007)
Scarlet eggplant	5	7.00	1.80	12.0	Dala‐Paula, Starling, et al. (2021)
Serralha ( *Sonchus oleraceus* L.)	3	nd	—	—	Dala‐Paula et al. (2024)
Spinach ( *Tetragonia expansa* )	10	0.80	0.60	1.10	Dala‐Paula, Starling, et al. (2021)
Tomato (*Lycopersicum esculentum* Mill.)	15	1.10	nd	3.10	Dala‐Paula, Starling, et al. (2021)
Tomato ( *L. esculentum* Mill.), cherry, conventional	4	3.30	—	—	Pinho, Almeida, et al. (2011)
Tomato ( *L. esculentum* Mill.), cherry, organic	4	9.50	—	—	Pinho, Almeida, et al. (2011)
*Mushroom*					
Mushroom, White Shimeji (*Pleurotus* spp.)	3	nd	—	—	Reis, Custódio, et al. (2020)
Mushroom, Hiratake (*Pleurotus* spp.)	3	nd	—	—	Reis, Custódio, et al. (2020)
Mushroom, Salmon (*Pleurotus* spp.)	3	nd	—	—	Reis, Custódio, et al. (2020)
Mushroom, Champignon (*Agaricus bisporus*)	5	nd	—	—	Reis, Guidi, et al. (2020)
Mushroom, Champignon (*A. bisporus*), cooked	2	nd	—	—	Reis, Dala‐Paula, et al. (2020)
Mushroom, Champignon, canned (*A. bisporus*)	2	nd	—	—	Reis, Dala‐Paula, et al. (2020)
Mushroom, Portobello (*A. bisporus*)	3	nd	—	—	Reis, Custódio, et al. (2020)
Mushroom, Shiitake (*Lentinula edodes*)	3	nd	—	—	Reis, Custódio, et al. (2020)
Mushroom, Black Shimeji (*Pleurotus* spp.)	3	nd	—	—	Reis, Custódio, et al. (2020)
Mushroom, Eryngii (*Pleurotus* spp.)	2	nd	—	—	Reis, Custódio, et al. (2020)
*Nuts*					
Almonds ( *Prunus dulcis* )	3	nd	—	—	Diniz (2015)
Brazil nuts ( *Bertholletia excelsa* )	3	nd	—	—	Diniz (2015)
Cashew nuts ( *Anacardium occidentale* )	3	nd	—	—	Diniz (2015)
Peanut ( *Arachis hypogaea* L.), roasted	5	nd	—	—	Diniz (2015)
*Pulses & derivatives*					
Cowpea					
Cowpea ( *Vigna unguiculata* L. Walp)	3	2.80	—	—	Cavalcante et al. (2019)
Cowpea ( *V. unguiculata* L. Walp)	12	nd	—	—	Barros et al. (2017)
Cowpea ( *V. unguiculata* L. Walp), cooked	12	nd	—	—	Barros et al. (2017)
Cowpea ( *V. unguiculata* L. Walp), cooked, broth	12	nd	—	—	Barros et al. (2017)
Beans					
Bean ( *Phaseolus vulgaris* L.), cooked	14	nd	—	—	Batista (2007)
Bean ( *Vigna radiata* L. R. Wilczek), sprout	10	28.8	4.9	87.5	Dala‐Paula, Starling, et al. (2021)
Soybean					
Soybean ( *Glycine max* L. Merril)	42	nd	—	—	Gloria et al. (2005)
Soybean ( *G. max* L. Merril), extract	10	nd	—	—	Batista (2007)
Soybean ( *G. max* L. Merril), protein concentrate	3	nd	—	—	Silva (2000)
Soybean ( *G. max* L. Merril), protein isolate	3	nd	—	—	Silva (2000)
Soybean ( *G. max* L. Merril), sprout	12	nd	—	—	Tavares Neto (2004)
*Spices & condiments*					
Tucupi	66	6.01	nd	66.9	Brito (2019)
Tucupi	3	2.26	—	—	Brito et al. (2019)
Tucupi	24	0.67	nd	1.33	Brito et al. (2022)
Tucupi	22	6.01	nd	66.9	Brito et al. (2023)
Soy sauce	42	123	nd	307	Guidi and Gloria (2012)
*Cocoa & chocolate*					
Cocoa ( *Theobroma cacao* ), fermented	9	nd	—	—	Deus et al. (2021)
Cocoa ( *T. cacao* ), fermented	9	nd	—	—	Brito et al. (2017)
Cocoa ( *T. cacao* ), fermented, roasted	6	nd	—	—	Konagano et al. (2022)
Cocoa ( *T. cacao* ), fermented, roasted	4	nd	—	—	Silveira et al. (2023)
Cocoa ( *T. cacao* ), fermented	4	nd	—	—	Silveira et al. (2023)
Coca ( *T. cacao* ), liquor	4	nd	—	—	Silveira et al. (2023)
Coca ( *T. cacao* ), liquor	4	nd	—	—	Silveira et al. (2023)
Chocolate, 60% cocoa	4	nd	—	—	Silveira et al. (2023)
Chocolate, 60% cocoa	4	nd	—	—	Silveira et al. (2023)
Chocolate, 70% cocoa	5	1.02	0.59	1.20	Silva et al. (2023)
Chocolate, 70% cocoa	27	1.70	nd	3.42	Deus et al. (2020)
Chocolate, 70% cocoa	3	nd	—	—	Dala‐Paula, Deus, et al. (2021)
*Coffee*					
Coffee, instant, powder	68	0.40	0.40	1.40	Silveira et al. (2007)
Coffee, instant, decaffeinated, beverage	50	nd	—	—	Silveira et al. (2007)
Coffee, instant, regular, beverage	30	nd	—	—	Silveira et al. (2007)
Coffee, soluble, organic, beverage	10	nd	—	—	Silveira et al. (2007)
Coffee ( *Coffea arabica* ), grain	8	nd	—	—	Oliveira (2004)
Coffee ( *C. arabica* ), grain	3	nd	—	—	Silveira (2008)
Coffee ( *C. arabica* ), grain	3	nd	—	—	Cirilo et al. (2003)
Coffee ( *C. arabica* ), defective green grain, black	3	4.00	—	—	Vasconcelos et al. (2007)
Coffee ( *C. arabica* ), defective green grain, immature	3	6.20	—	—	Vasconcelos et al. (2007)
Coffee ( *C. arabica* ), defective green grain, sour	3	9.20	—	—	Vasconcelos et al. (2007)
Coffee ( *C. arabica* ), non‐defective green grain	3	nd	—	—	Vasconcelos et al. (2007)
Coffee ( *C. arabica* ), grain, roasted	8	nd	—	—	Oliveira (2004)
Coffee ( *C. arabica* ), non‐defective grain, light roast	12	nd	—	—	Vasconcelos et al. (2007)
Coffee ( *C. arabica* ), grain, roasted	3	nd	—	—	Silveira (2008)
Coffee ( *C. arabica* ), grain, American roast	3	nd	—	—	Cirilo et al. (2003)
Coffee ( *C. arabica* ), grain, French roast	3	nd	—	—	Cirilo et al. (2003)
*Cereals & derivatives*					
Corn ( *Zea mays* L.)					
Corn, sweet	3	nd	—	—	Bandeira et al. (2012)
Corn, sweet, conventional	80	nd	—	—	Pinho, Paes, et al. (2011)
Corn, sweet, organic	80	nd	—	—	Pinho, Paes, et al. (2011)
Corn, canned	3	nd	—	—	Bandeira et al. (2012)
Corn, dried	12	1.06	nd	2.11	Bandeira et al. (2012)
Corn, meal	10	nd	—	—	Batista (2007)
Corn, endosperm	3	nd	—	—	Bandeira et al. (2012)
Corn, embryo	3	nd	—	—	Bandeira et al. (2012)
Corn, starch	10	2.3	—	—	Batista (2007)
Corn, sprout	10	1.13	—	—	Bandeira et al. (2012)
Sorghum ( *Sorghum bicolor* L.)					
Sorghum, tannin	10	nd	—	—	Paiva et al. (2015)
Sorghum, no tannin	12	nd	—	—	Paiva et al. (2015)
Sorghum, tannin, germinated	4	nd	—	—	Paiva et al. (2022)
Sorghum, no tannin, germinated	4	nd	—	—	Paiva et al. (2022)
Rice					
Rice, cooked	14	nd	—	—	Batista (2007)
*Alcoholic beverages*					
Wine					
Wine, white	2	0.28	—	—	Sabaini (2009)
Wine, white	15	1.64	—	—	Agustini et al. (2014)
Wine, white	3	0.08	0.01	0.23	Camargo (2022)
Wine, Cabernet Franc	6	0.49	—	—	Souza et al. (2005)
Wine, Cabernet Sauvignon	8	0.92	0.23	1.73	Souza et al. (2005)
Wine, Cabernet Sauvignon	6	3.80	3.79	3.80	Rossato (2005)
Wine, Merlot	3	0.61	0.07	1.67	Souza et al. (2005)
Wine, Merlot	12	nd	—	—	Manfroi et al. (2009)
Wine, Rose	3	nd	—	—	Sabaini (2009)
Wine, Rose	4	0.57	0.01	2.23	Camargo (2022)
Wine, Syrah	3	1.69	—	—	Mota et al. (2009)
Wine, Syrah	7	nd	—	—	Nassur et al. (2017)
Wine, red	6	1.98	—	—	Sabaini (2009)
Wine, red, dry	19	3.50	—	—	Agustini et al. (2014)
Wine, red, dry	8	0.31	0.02	0.92	Camargo (2022)
Wine, red, sweet	13	2.40	—	—	Agustini et al. (2014)
Wine, red, sweet	2	0.13	0.07	0.19	Camargo (2022)
Wine, table	3	1.53	—	—	Daniel et al. (2015)
Wine, table	45	5.03	nd	8.77	R. F. Braga (2015)
Wine, table, white	3	0.53	—	—	Lourenço et al. (2022)
Wine, xisto	63	2.52	0.60	4.50	Colimo (2013)
Wine, sparkling	5	0.40	—	—	Sabaini (2009)
Beer					
Beer, alcohol free	7	0.16	nd	0.62	Gloria and Izquierdo‐Pulido (1999)
Beer, bock	23	0.29	nd	1.46	Gloria and Izquierdo‐Pulido (1999)
Beer, pale ale	2	nd	—	—	Botelho (2009)
Beer, pale ale	2	1.04	—	—	Daniel et al. (2015)
Beer, ice	5	nd	—	—	Gloria and Izquierdo‐Pulido (1999)
Beer, extra	2	nd	—	—	Botelho (2009)
Beer, lager	46	0.17	nd	0.90	Gloria and Izquierdo‐Pulido (1999)
Beer, lager	2	0.45	—	—	Daniel et al. (2015)
Beer, lager	4	nd	—	—	Botelho (2009)
Beer, Pilsen	14	nd	—	—	Botelho (2009)
Beer, Pilsen	2	0.75	—	—	Daniel et al. (2015)
Beer, Malzbier	6	nd	—	—	Botelho (2009)
Beer, Stout	8	nd	—	—	Botelho (2009)
Beer, Stout	10	0.25	nd	0.85	Gloria and Izquierdo‐Pulido (1999)
Liquor					
Liquor, Jabuticaba	6	nd	—	—	Neves et al. (2022)
*Miscellaneous*					
Oils					
Soybean oil	10	nd	—	—	Batista (2007)
Olive oil	10	nd	—	—	Batista (2007)
Sweets					
Sugar	10	nd	—	—	Batista (2007)
Seaweed					
Seaweed (Chlorophyta, Rhodophyta, Phaeophyta) dried	39	0.01	nd	0.01	Alencar et al. (2011)
Extracts					
Eggplant ( *Solanum melongena* L.), extract	3	386	—	—	Botelho et al. (2004)
*Bauhinia holophylla* dried leaf aqueous extract	3	0.50	—	—	Savazzi et al. (2024)

*Note:* nd, not detected (below detectable level ≤ 1.0 mg/kg). (—) not available.

### 7.1 Histamine Levels in Food of Animal Origin

HIM was detected in every food group of animal origin (Table 3), which suggests that they are prone to HIM formation and build‐up. This is probably because they are generally rich in proteins, which can undergo proteolysis during storage, processing, fermentation, or deterioration. Proteolysis can release histidine, which can be decarboxylated into HIM (Gloria 2005; Ruiz‐Capillas and Herrero 2019; Simon Sarkadi 2019).

When considering Milk & Derivatives, HIM was not detected in good‐quality raw cow's milk, as reported in the literature (Moniente et al. 2021; Ferrante and Mercogliano 2023); however, it was present in sheep's milk. HIM was detected, at low levels, in pasteurized, fermented milk and dairy beverages. Still, these products could be a source of HIM, depending on the quality of the milk and other ingredients, the type of microorganisms added (starters), the fermentation and processing conditions, good manufacturing practices, and the storage temperature and duration (Schirone, Visciano, et al. 2022; Ferrante and Mercogliano 2023). HIM was detected at low levels in cheese whey, the raw material for many dairy beverages. When considering non‐aged cheese (mozzarella, fresh Minas, requeijão, and fresh artisanal), HIM levels were ≤ 64.4 mg/kg. HIM increases with cheese aging, reaching levels ≤ 423 mg/kg, as indicated in the literature (Benkerroum 2016; Moniente et al. 2021; Ferrante and Mercogliano 2023). Grated cheese showed the highest HIM levels (≤ 1154 mg/kg), as attested by EFSA (2011). This is probably due to poor‐quality raw material. In some countries, defective cheese is allowed as an ingredient for grated cheese, without defining quality standards. The HIM content in dairy products correlates significantly with milk quality, hygienic conditions during processing, aging, and storage period and conditions (Benkerroum 2016; Moniente et al. 2021; Ferrante and Mercogliano 2023). HIM can accumulate in cheese during the metabolism of starter and non‐starter lactic acid bacteria, yeasts, and spoilage bacteria (Moniente et al. 2021). Since there were samples with no detectable HIM levels in several dairy products, producing products with no detectable HIM is possible. This can be ensured by using good manufacturing practices and selecting starters free of histidine decarboxylase (Ferrante and Mercogliano 2023).

Fresh, good‐quality, and well‐kept meats—beef, pork, poultry, and alligator—had low HIM levels, as Schirone, Esposito, et al. (2022) and Wójcik et al. (2022) reported. However, Lázaro et al. (2014) found high HIM in chicken breast, probably due to poor hygienic and storage conditions. Meat derivatives (corned beef, hamburger, meatballs, mortadella, and nuggets) had increased HIM levels. Dry‐fermented sausages had the highest HIM, in consonance with the literature (Latorre‐Moratalla et al. 2017; Halagarda and Wójciak 2022; Schirone, Esposito, et al. 2022; Turna et al. 2024; Braga et al. 2025). The highest HIM levels were found in Hamburgues and Italian gourmet dry‐fermented sausages. Many factors can contribute to HIM accumulation in meat products, including the quality of the raw materials, other ingredients added (e.g., sugar), hygienic practices during processing, manufacturing steps and conditions, fermentation, ripening, the quality and quantity of the microbial flora, and the use of starter culture (Latorre‐Moratalla et al. 2017; Halagarda and Wójciak 2022; Schirone, Esposito, et al. 2022; Turna et al. 2024; Braga et al. 2025). To prevent HIM formation during fermentation, the selection of starter cultures without histidine decarboxylase activity, hygienic practices, and temperature control are required (Comas‐Basté et al. 2019; Turna et al. 2024).

For Fish and Derivatives, HIM was not detected in 50% of the fresh or frozen fish. This is probably due to the freshness of the fish, storage at low temperatures (< 4°C), and good hygienic practices during capture, transport, processing, distribution, and storage conditions (Comas‐Basté et al. 2019; Arulkumar et al. 2023; Ding and Li 2024). Fish families can be categorized into groups according to their susceptibility to produce HIM (EC 2005; FDA 2011; Brasil 2021). Among fish from the susceptible families, those from Carangidae, Gempylidae, Istiophoridae, Clupeidae, Engraulidae, Coryfenidae, Pomatomidae, Scombridae, and Scombresosidae families stand out. HIM is the only biogenic amine with regulatory limits for susceptible fish set by legislation worldwide (EC 2005; FDA 2011). A maximum safe level of 200 mg/kg was suggested (FAO/WHO 2018). However, country‐by‐country regulations vary from 50 to 400 mg/kg (DeBeer et al. 2021). In Brazil, the limit is 100 mg/kg (Brasil 2021). Most of the HIM levels in fish complied with Brazilian and international legislations, except for 14 samples (1.0%), e.g., seven tunas, four sardines, and three tainhas. Among the non‐susceptible fish, the higher mean and maximum HIM levels were observed for tainha (134 mg/kg) in fish from the municipal market, suggesting poor hygienic conditions and temperature control. Among fish from susceptible families, higher HIM was found in tuna (649 mg/kg), followed by sardine (584 mg/kg). Canned tuna and sardines had HIM levels complying with legislation. However, higher HIM were found in grated fish (higher surface area), and fish with other ingredients. High HIM levels were found in salmon sashimi, probably due to handling and poor temperature control. Regarding crustaceans, HIM was detected at low levels in shrimp but high levels in crabs. However, Arulkumar et al. (2023) reported low HIM in shrimp and crab (~10 mg/kg), which deserves further investigation.

Eggs are a poor source of HIM, which has been confirmed by Ramos and Ferreira (2009)—the fresher the eggs, the lower the HIM levels. HIM levels are lower in egg yolks than in egg whites. Honey was claimed to have HIM. However, Jochum (2024) indicated that honey is a poor source of HIM, warranting further investigation.

### 7.2 Histamine Levels in Food of Plant Origin

Foods of plant origin were stratified into twelve groups (Table 4). In the Fruits group, 30 types were included, some from different varieties. HIM was not detected in most fruits (87%), but it was found at levels ≤ 2.80 mg/kg in a few, including grapes, melons, oranges, peaches, and watermelons. Bergamot was the fruit with the highest HIM. The presence of HIM in grapes has been reported previously (Ordóñez et al. 2016; Incesu et al. 2022). However, according to Sánchez‐Pérez et al. (2018), HIM was not detected in oranges and peaches. Some fruits were confirmed to have no detectable HIM, including apples (O'Sullivan 2000; Ordóñez et al. 2016; Preti et al. 2016), lemons, passion fruits (Sánchez‐Pérez et al. 2018), avocados, bananas, strawberries (Sánchez‐Pérez et al. 2018, 2021), papayas (Sánchez‐Pérez et al. 2021), and pineapples (Sánchez‐Pérez et al. 2018, 2021). Studies on HIM in açai berries, acerola, custard apple, genipap, guava, jabuticaba, khaki, marolo, mango, pear, seriguela, soursop, star fruit, and umbu are available for the first time, and all had no detectable HIM.

In Fruit Derivatives, HIM was detected in orange juice, as Preti et al. (2016) and Sánchez‐Pérez et al. (2018) reported. It was also detected in orange soft drinks containing 10% orange juice (Vieira et al. 2007). Sixty‐three percent of the grape juices and musts had HIM. Similar results were reported in the literature (La Torre et al. 2023). HIM is also found in grape peel flour.

HIM was detected in 37.5% of the 24 types of Vegetables investigated. Eggplants had the highest HIM mean levels, followed by tomatoes, scarlet eggplants (all from the Solanaceae family), and pumpkins. Some other vegetables had lower HIM levels, including beets, spinach, sweet potatoes, and cauliflowers. Previous studies have confirmed the presence of high levels of HIM in eggplants (Sánchez‐Pérez et al. 2018, 2021; Comas‐Basté et al. 2020). According to Dala‐Paula, Starling, et al. (2021), HIM levels were higher in the eggplant peel, followed by the core, whereas the level in the pulp was lower. HIM has also been reported in tomatoes (Sánchez‐Pérez et al. 2018, 2021; Comas‐Basté et al. 2020), pumpkins (Sánchez‐Pérez et al. 2018), and spinach (Sánchez‐Pérez et al. 2018; Comas‐Basté et al. 2020; Yilmaz and Gökmen 2020; Sánchez‐Pérez et al. 2021). The occurrence of HIM in cauliflowers contradicts the findings of Sánchez‐Pérez et al. (2018). Some vegetables had no detectable HIM, such as lettuce, onions (Sánchez‐Pérez et al. 2018); potatoes (O'Sullivan 2000); and carrots (O'Sullivan 2000; Sánchez‐Pérez et al. 2018). Information on capers, cassava, chayote, collard greens, heart‐of‐palm, kale, onion greens, ora‐pro‐nobis, potatoes, and parsley was provided for the first time.

Mushrooms did not have detectable HIM, which was confirmed by Sánchez‐Pérez et al. (2018, 2021) and Dadáková et al. (2022). Similarly, nuts, including almonds, Brazil nuts, cashew nuts, and peanuts, did not have detectable HIM. Results were confirmed for peanuts and almonds (Sánchez‐Pérez et al. 2018, 2021). Cashew and Brazil nuts were analyzed for the first time.

Among the Pulses and Derivatives group, HIM was detected in cowpeas. However, soybeans and products and beans had no detectable HIM, as attested by Preti et al. (2017) and Sánchez‐Pérez et al. (2018, 2021). However, bean sprouts had high HIM. The moist environment, typical of germination, during bean sprout production, allows microbial growth, increasing the possibility of HIM formation by microbial amino acid decarboxylase activity (Dala‐Paula, Starling, et al. 2021).

The Spice and Condiments group had the highest HIM levels. Two types of products were analyzed. Soy sauce had the highest levels (≤ 307 mg/kg), as confirmed in the literature (Ordóñez et al. 2016; Yilmaz and Gökmen 2020; Turna et al. 2024). Tucupi, a traditional and popular fermented condiment in the Northern Brazilian diet (Brito et al. 2019), was also a source of HIM, varying from not detected to 66.9 mg/kg.

In the Cocoa and Chocolate group, HIM was found sporadically in 60% and 70% chocolate at levels ≤ 3.42 mg/kg, as described in the literature (Ordóñez et al. 2016; Sánchez‐Pérez et al. 2018; Yilmaz and Gökmen 2020; Dabadé et al. 2021). However, HIM was not detected in cocoa and cocoa liquor, suggesting that the ingredients used in chocolate could be the source of HIM. HIM was not detected in good‐quality coffee in the Coffee group, except in defective grains (black, sour, and green), which showed ≤ 9.2 mg/kg HIM levels. The presence of HIM in green coffee has also been reported by Yilmaz and Gökmen (2020).

The Cereals & Derivatives group's foods were low in HIM. Sánchez‐Pérez et al. (2018) confirmed that sweet corn and rice had no detectable HIM. However, HIM was detected in dried corn and corn starch because of contamination during processing (Sánchez‐Pérez et al. 2018). In addition, HIM was also found in corn sprouts, probably because of the germination conditions.

For Alcoholic Beverages, higher HIM levels were found in wines compared to beer. HIM has been widely reported in wine (Yilmaz and Gökmen 2020; La Torre et al. 2023). Table wine had higher mean levels than red and white wines (EFSA 2011; Yilmaz and Gökmen 2020). Beers were lower in HIM than wines (Yilmaz and Gökmen 2020).

The Miscellaneous group included oils, sweets, seaweed, and plant extracts. Although olives are known to be rich in HIM (Dabadé et al. 2021), HIM was not detected in olive oil. Nor was it detected in soybean oil. HIM was not detected in cane sugar. Seaweed (Alencar et al. 2011) had low HIM levels. High HIM levels were found in eggplant extract, which is commonly used to control blood cholesterol levels. This finding warns against the product's use, as there is no scientific evidence to support a benefit, especially for individuals with HIM intolerance. *Bauhinia holophylla* dried leaf aqueous extract, popularly used for its hypoglycemic effect, also contained HIM.

## 8 Risk Assessment Associated With Histamine Levels in Brazilian Food

As described previously, DAO is the primary enzyme that metabolizes ingested HIM and is responsible for scavenging extracellular HIM (Dala‐Paula et al. 2023; Rentzos et al. 2024). However, excessive HIM levels in foods and factors affecting HIM degradation, including DAO inhibition, can result in excess HIM, triggering HIM intoxication or intolerance. The NOAEL after exposure to HIM was reported to be 50 mg HIM per person per meal for HIM intoxication, but below detectable levels for individuals with HIM intolerance (EFSA 2011, 2017). In this way, HIM levels above 50 mg and any detectable level per person per meal could trigger HIM intoxication and intolerance, respectively.

**FIGURE 4:**
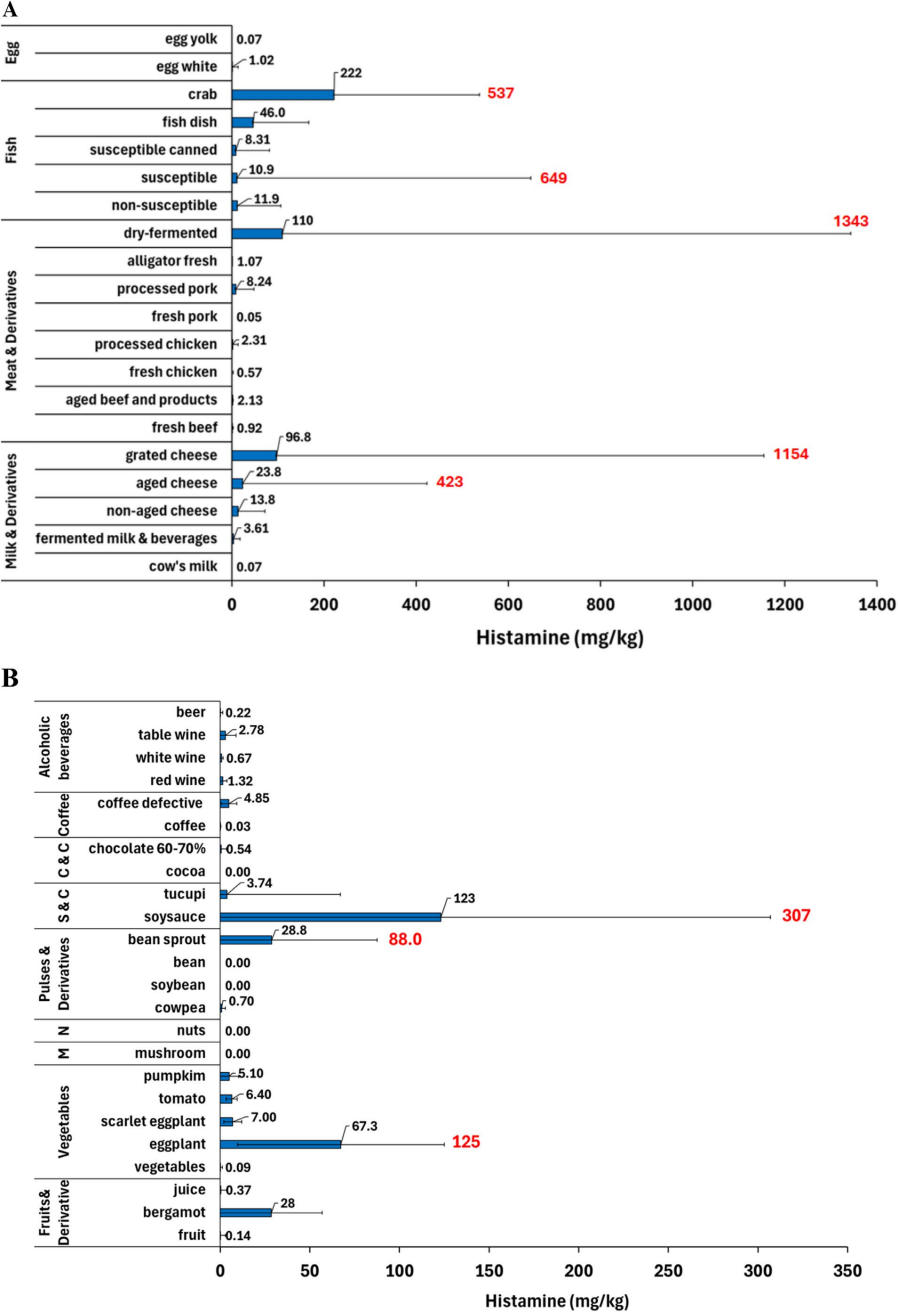
Mean levels of histamine in different types of food of (A) animal and (B) plant origins. C & C—Cocoa & Chocolate. S & C—Spice & Condiments. N—Nuts. M—Mushrooms. Values in red are maximum levels.

Mean HIM levels were calculated for each group of food products and for special food products using the lower‐bound approach, e.g., assigning zero to samples reported as no detectable—nd (< LOQ). These values are provided in Figure 4. In addition, the maximum HIM levels (worst‐case scenario) were presented for specific foods of animal and plant origin.

When considering the likelihood of causing HIM intoxication, several products deserve attention and should be consumed with special care and moderation. For example, when considering some food at their highest level, NOAEL (50 mg HIM) could be reached by consuming either 37.2 g of dry‐fermented sausage, 43.3 g of grated cheese, 77 g of tuna (Scombridae), 118.2 g of aged cheese, 299 g of fish dish, 163 mL of soy sauce, or 400 g of eggplant. One must also consider that the combination of HIM‐containing food would further contribute to total levels. Another approach that could be used is to compare the HIM levels in the foods with those reported in HIM intoxication episodes (> 422 mg/kg). This way, dry‐fermented sausage, grated cheese, tuna, and aged cheese could lead to HIM intoxication. Indeed, tuna (Scombridae) and other fish susceptible to HIM formation have been widely associated with HIM intoxication episodes (EFSA 2011, 2017; Colombo et al. 2018; Takemoto et al. 2014, 2019). Sporadic outbreaks have been reported with aged cheeses such as Gouda, Swiss, grated cheese (Tsakona 2015; Colombo et al. 2018), and crab (Yu et al. 2018). Interesting that, although dry‐fermented sausages are rich in HIM (EFSA 2011; Latorre‐Moratalla et al. 2017; Halagarda and Wójciak 2022; Schirone, Esposito, et al. 2022; Turna et al. 2024; Braga et al. 2025), little information is available regarding their involvement in HIM intoxication, which deserves further studies.

The risk of HIM intolerance is much higher, considering that even small amounts of HIM may trigger adverse effects. Most products of animal origin are of concern unless they are from reliable sources, fresh, of good quality, stored under adequate conditions, and not processed or fermented. Dry‐fermented sausages and aged and grated cheese should be avoided because of the high HIM levels. According to Sánchez‐Pérez et al. (2021), of the ten reports on HIM‐free diets, all recommended avoiding these products, and 90% recommended avoiding fish to prevent HIM intolerance. Fresh, good‐quality products of plant origin are usually safe, whereas inadequate processing, storage, fermentation, and germination can increase HIM levels. Special care must be taken when using fermented products (soy sauce, tucupi, and fermented beverages), as they may contain high HIM levels. According to Sánchez‐Pérez et al. (2021), products of plant origin that should be avoided to prevent HIM intolerance include wine and beer (100% of the studies), tomatoes (90%), spinach (80%), and sauerkraut (60%). Therefore, recommendations to avoid these products are appropriate. Some HIM‐containing fruits and vegetables that should be avoided include bergamot, grapes, melons, oranges, peaches, watermelons, beets, cauliflowers, eggplants, scarlet eggplants, sweet potatoes, pumpkins, bean sprouts, and other germinated products, cowpeas, and defective coffee beans. Sweet potatoes (El‐Qutob et al. 2018) and beets (Oliveira et al. 2011) have elicited HIM intolerance‐like symptoms, including urticaria and asthma. Nevertheless, some foods of plant origin were confirmed to contain HIM below detection limits, and their ingestion may be safe for individuals with HIM intolerance.

### 8.1 Prevention of Histamine Intoxication and Intolerance

**FIGURE 5:**
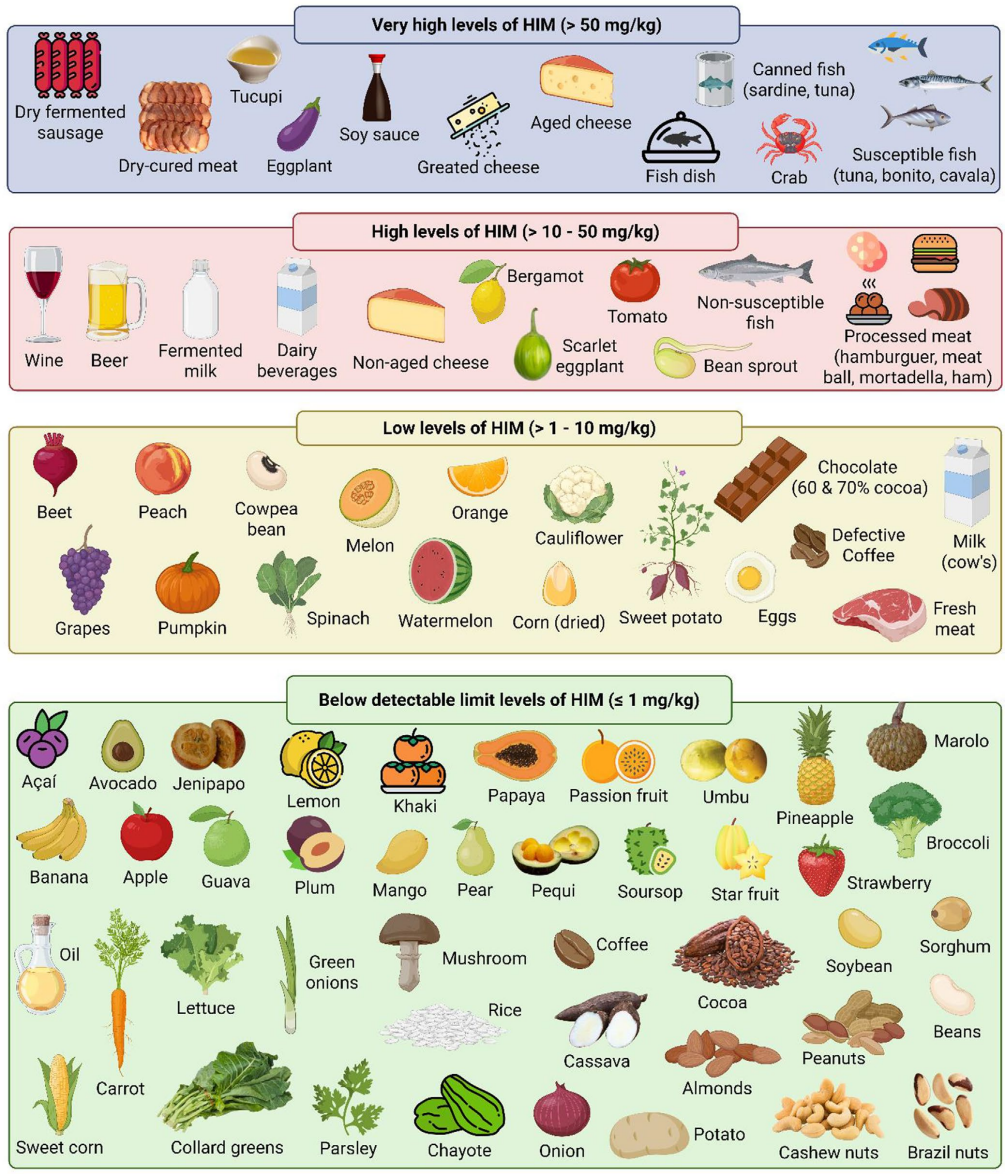
Classification of food from the Brazilian diet according to the occurrence and levels of histamine (HIM): Below detectable limit levels of HIM (≤ 1 mg/kg). Low levels of HIM (> 1 to 10 mg/kg). High levels of HIM (> 10 to 50 mg/kg). Very high HIM (> 50 mg/kg). Susceptible fish: Fish from families Carangidae, Gempylidae, Istiophoridae, Clupeidae, Engraulidae, Coryfenidae, Pomatomidae, Scombridae, and Scombresosidae.

To prevent HIM intoxication and intolerance, it is essential to be aware of the occurrence of HIM in food. Based on the scientific information provided (Tables 3 and 4, and Figure 4), the foods were categorized, according to the respective HIM levels (Figure 5), in four major groups: below detectable limits (< 1.0 mg/kg), low (1.0 to 10 mg/kg), high (10 to 50 mg/kg), and very high levels (> 50 mg/kg). It is crucial to consider that, for NOAEL, all meal components must be considered, and the total HIM must be calculated. Therefore, some food combinations, such as wine and cheese, tuna with eggplant, should also be avoided. Another critical point is that heat treatment does not diminish HIM levels because HIM is heat‐stable (Gloria 2005).

To prevent HIM intoxication, one must be aware that fish, especially from the Scombridae family (and other susceptible ones), and grated and aged cheese are the most often incriminated. Therefore, these products must be purchased from reliable sources before expiration and kept under adequate hygienic, sanitary, and storage conditions. Fish must be fresh, of good quality, and kept refrigerated (< 4°C) or frozen. Frozen fish should be thawed at a refrigerated temperature. Refreezing should be avoided. Purchasing and grating the cheese yourself is advisable (Gloria et al. 2005).

The most advised strategy for preventing HIM intolerance is to adhere to low‐HIM or HIM‐free diets, avoiding HIM‐containing foods (Sánchez‐Pérez et al. 2022; Vidal‐Carou et al. 2022; Dala‐Paula et al. 2023; Zingone et al. 2023). According to Figure 5, it is safe to consume food items with HIM levels below detectable limits (< 1 mg/kg). Food with medium and high HIM must be avoided. Individuals following a low‐HIM diet can use, with moderation, food from the group with low HIM (1 to 10 mg/kg). However, fresh and good‐quality food should always be chosen over stored or highly elaborated and preserved foods.

Another strategy to prevent HIM intolerance is enzyme therapy, for example, supplementation with exogenous DAO to improve digestion and enhance intestinal HIM degradation (Sánchez‐Pérez et al. 2021; Jochum 2024; Rentzos et al. 2024). The main objective is to prevent symptoms and resolve clinical manifestations associated with HIM intolerance (Zingone et al. 2023). However, this requires a more rigorous evaluation (Jochum 2024). Recent speculation regarding the potential of mast cell stabilizers (Zingone et al. 2023) also deserves further studies.

## 9 Concluding Remarks and Perspectives

The most recent findings on HIM biochemistry and the health effects associated with HIM are summarized, providing up‐to‐date information on the occurrence, symptoms, and strategies to prevent HIM intoxication and intolerance.

The systematic literature review (1980 to 2024) led to 156 documents on the HIM levels in foods from the Brazilian diet. Altogether, reliable HIM levels were provided for 343 types of food. The foods were categorized into four groups regarding HIM levels: below detectable limits, low, high, and very high. Many uncertainties and doubts about the occurrence of HIM in foods on social media were clarified. However, studies must continue to broaden the types of foods analyzed for HIM. This is relevant because long‐term adherence to a restrictive diet with a limited number of food choices poses additional challenges of compliance, difficulty in meeting nutritional needs, and increased risk of developing unhealthy eating patterns.

The reliable and scientific data provided on HIM occurrence in food will reassure individuals regarding food choices, prevent HIM intoxication, and contribute to an improved quality of life for individuals with HIM intolerance. It will also enable healthcare professionals to prescribe adequate low‐HIM or HIM‐free diets based on scientific evidence. Therefore, accurate data on foods' HIM levels is essential for clinical nutrition and public health strategies. For dietitians, such information enables the development of targeted dietary interventions, especially for individuals with HIM intolerance.

From a public health policy and risk management perspective, decision makers within the food industry and governmental agencies should consider actions to reduce consumer exposure to dietary HIM. For example, regulatory measures or strategies could be implemented to prevent, reduce, or eliminate HIM and other amines in foods (Comas‐Basté et al. 2020). Another way to ensure safety is the label declaration of HIM occurrence or absence by the food industry, which could help individuals with HIM intolerance make suitable, safe, and informed choices (Sánchez‐Pérez et al. 2021).

However, this study raised several questions that warrant further investigation. Therefore, we highlight some identified research gaps and future directions needed to improve the knowledge about HIM in foods and the inherent adverse effects.

First, analysis must continue regarding the occurrence and levels of HIM and other amines in food. Although this study provided histamine levels for 343 different types of food, it does not encompass the diversity of food products in the Brazilian and world markets.

Although dry‐fermented sausages have the highest HIM content, their association with HIM intoxication has rarely been reported. Therefore, studies are needed to understand why high HIM levels in these products are not associated with HIM intoxication. An approach would be to undertake in vitro digestion of this and other peculiar foods.

Another question is related to reports about foods that can trigger the release of HIM from mast cells. However, scarce information on the mechanism responsible for this potential effect is available. Additionally, no consensus exists on which foods are HIM‐releasers (Vlieg‐Boerstra et al. 2005; Sánchez‐Pérez et al. 2021; Shulpekova et al. 2021).

An additional area that deserves further study is the simultaneous occurrence of other diamines, e.g., PUT and CAD, along with HIM, and their possible interference in HIM degradation by DAO in the intestinal tract (Sánchez‐Pérez, Comas‐Basté, Costa‐Catala, et al. 2022; Zingone et al. 2023). These biogenic amines might interfere with histamine degradation by DAO at the intestinal level (Sánchez‐Pérez et al. 2021). Nevertheless, limited experimental evidence supports this hypothesis (Zingone et al. 2023). The role of ethanol in HIM degradation by DAO in the intestinal tract also deserves investigation.

Another topic for additional scrutiny is associated with recent studies suggesting that HIM intolerance can arise from an imbalance or dysbiosis of the gut microbiota (Zingone et al. 2023). According to Sánchez‐Pérez, Comas‐Basté, Duelo, et al. (2022), there is a higher abundance of HIM‐secreting (histaminogenic) bacteria, including the genera *Staphylococcus* and *Proteus*, and some Enterobacteriaceae genera in individuals with HIM intolerance, in contrast to the high proportion of Prevotellaceae, Ruminococcus, Faecalibacterium, and *Faecalibacterium prausnitzii* associated with a healthy gut. The higher abundance of histaminogenic bacteria in the gut can result in an excess accumulation and systemic absorption of histamine (Sánchez‐Pérez, Comas‐Basté, Duelo, et al. 2022), which requires further studies.

## Author Contributions

**Maria Beatriz A. Gloria:** conceptualization (lead), funding acquisition (lead), project administration (lead), supervision (lead), writing – original draft (lead). **Fabiana B. Diniz:** conceptualization (equal), data curation (equal), formal analysis (equal), investigation (equal), methodology (equal), writing – review and editing (equal). **Bruno M. Dala‐Paula:** investigation (supporting), methodology (equal), validation (equal), writing – review and editing (equal). **Biane Philadelpho:** data curation (equal), investigation (equal), methodology (equal), resources (equal). **José Eduardo Gonçalves:** conceptualization (equal), formal analysis (equal), methodology (equal), supervision (equal). **Ederlan S. Ferreira:** data curation (equal), formal analysis (equal), methodology (equal), writing – review and editing (equal). **Livia Simon Sarkadi:** data curation (equal), investigation (equal), methodology (equal), writing – review and editing (equal).

## Conflicts of Interest

The authors declare no conflicts of interest.

## Data Availability

The data that support the findings of this study are openly available in the Digital Library of Theses and Dissertations (BDTD) from IBCTI (https://bdtd.ibict.br/vufind/), the portal of publications and scientific data in open access (AOSISBR) (https://oasisbr.ibict.br/vufind/), and the CAPES catalog (http://capesdw.capes.gov.br/).
